# Efficacy and Mechanism of Action of Low Dose Emetine against Human Cytomegalovirus

**DOI:** 10.1371/journal.ppat.1005717

**Published:** 2016-06-23

**Authors:** Rupkatha Mukhopadhyay, Sujayita Roy, Rajkumar Venkatadri, Yu-Pin Su, Wenjuan Ye, Elena Barnaeva, Lesley Mathews Griner, Noel Southall, Xin Hu, Amy Q. Wang, Xin Xu, Andrés E. Dulcey, Juan J. Marugan, Marc Ferrer, Ravit Arav-Boger

**Affiliations:** 1 Department of Pediatrics, Division of Infectious Diseases, Johns Hopkins University School of Medicine, Baltimore, Maryland, United States of America; 2 National Center for Advancing Translational Sciences, National Institutes of Health, Rockville, Maryland, United States of America; University of Wisconsin-Madison, UNITED STATES

## Abstract

Infection with human cytomegalovirus (HCMV) is a threat for pregnant women and immunocompromised hosts. Although limited drugs are available, development of new agents against HCMV is desired. Through screening of the LOPAC library, we identified emetine as HCMV inhibitor. Additional studies confirmed its anti-HCMV activities in human foreskin fibroblasts: EC_50_−40±1.72 nM, CC_50_−8±0.56 μM, and selectivity index of 200. HCMV inhibition occurred after virus entry, but before DNA replication, and resulted in decreased expression of viral proteins. Synergistic virus inhibition was achieved when emetine was combined with ganciclovir. In a mouse CMV (MCMV) model, emetine was well-tolerated, displayed long half-life, preferential distribution to tissues over plasma, and effectively suppressed MCMV. Since the *in vitro* anti-HCMV activity of emetine decreased significantly in low-density cells, a mechanism involving cell cycle regulation was suspected. HCMV inhibition by emetine depended on ribosomal processing S14 (RPS14) binding to MDM2, leading to disruption of HCMV-induced MDM2-p53 and MDM2-IE2 interactions. Irrespective of cell density, emetine induced RPS14 translocation into the nucleus during infection. In infected high-density cells, MDM2 was available for interaction with RPS14, resulting in disruption of MDM2-p53 interaction. However, in low-density cells the pre-existing interaction of MDM2-p53 could not be disrupted, and RPS14 could not interact with MDM2. In high-density cells the interaction of MDM2-RPS14 resulted in ubiquitination and degradation of RPS14, which was not observed in low-density cells. In infected-only or in non-infected emetine-treated cells, RPS14 failed to translocate into the nucleus, hence could not interact with MDM2, and was not ubiquitinated. HCMV replicated similarly in RPS14 knockdown or control cells, but emetine did not inhibit virus replication in the former cell line. The interaction of MDM2-p53 was maintained in infected RPS14 knockdown cells despite emetine treatment, confirming a unique mechanism by which emetine exploits RPS14 to disrupt MDM2-p53 interaction. Summarized, emetine may represent a promising candidate for HCMV therapy alone or in combination with ganciclovir through a novel host-dependent mechanism.

## Author Summary

Infection with human Cytomegalovirus (HCMV) is a growing and pressing problem, creating ongoing management and therapeutic challenges. Despite the availability of DNA polymerase inhibitors, development of new strategies for HCMV therapy is needed. We report for the first time on the efficacy of an old drug (emetine) against HCMV *in vitro* and mouse CMV *in vivo*, using exceedingly low drug doses. We also provide evidence for a specific host-dependent anti-CMV mechanism of emetine *in vitro*, thus uncovering a cellular function that can be further studied for drug development. Our work provides a novel direction for HCMV therapeutics through repurposing of an old agent, at substantially lower doses, and inhibiting HCMV indirectly through host activities critical for virus replication.

## Introduction

Infection with Human Cytomegalovirus (HCMV) continues to be a major threat for transplant recipients and patients with AIDS [1–3]. It is also the most common congenital infection worldwide [4]. The systemic anti-HCMV drugs all target the viral DNA polymerase and effectively suppress HCMV replication [5]. However, their use is associated with toxicities to the bone marrow (ganciclovir-GCV) and kidneys (foscarnet and cidofovir) [6,7]. GCV and its oral formulation val-GCV are the only drugs used for congenital HCMV infection, based on improved hearing and neurodevelopmental outcomes achieved in infected children [8,9]. Because of the limited drugs approved for HCMV, the side effects associated with them, and the emergence of resistant viral mutants during therapy [7,10,11], there is a pressing need to develop anti-HCMV compounds with novel mechanisms of action. HCMV inhibitors that target viral proteins other than the DNA polymerase have been identified; UL97 kinase inhibitor, maribavir [12,13] and the terminase inhibitor, letermovir [14,15]. These agents are in different stages of development [14–16], and because of the direct virus targets they also select for virus mutants.

As part of developing new therapeutics for HCMV, deciphering its complex and evolving interaction with the cellular machinery is necessary for identification of new targets required for HCMV replication. Towards this goal, we screened a library of pharmacologically active compounds (LOPAC1280) and identified emetine as potential HCMV inhibitor. We report on the *in vitro* anti-HCMV activities of emetine, *in vivo* activities in a mouse CMV (MCMV) model, and a novel host-dependent anti-viral mechanism of HCMV inhibition.

## Results

### Emetine inhibits HCMV and HSV replication at nM concentrations

**Fig 1:**
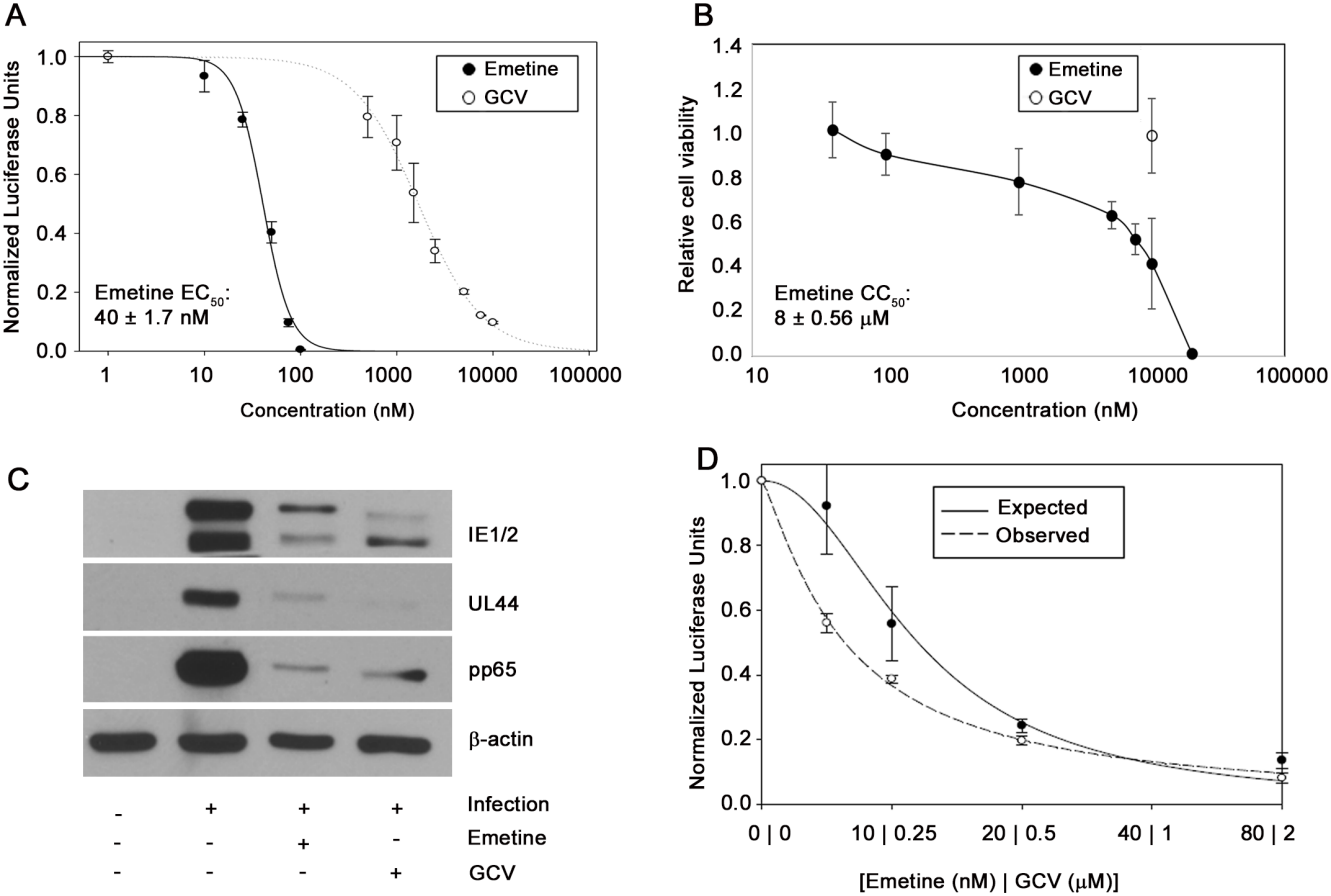
Anti-HCMV activity of emetine and synergy with GCV. **A)** Cells were infected with pp28-luciferase HCMV Towne and treated with indicated concentrations of emetine or GCV. Luciferase activity was measured at 72 hpi. Data represent mean ± SE of triplicate determinations from a representative of three independent experiments. **B)** Cells were treated with the indicated concentrations of emetine and cell viability was determined after 72 h. Data represent mean values ± SE of triplicate determinations from a representative of three independent experiments. **C)** Inhibition of HCMV protein expression by emetine (75 nM) or GCV (5 μM) at 72 hpi. Cells were infected with HCMV Towne and treated with emetine. Western blot of viral proteins IE1/2, UL44 and pp65 was performed at 72 hpi. **D)** Cells were infected with pp28-luciferase HCMV Towne, and treated with combination of GCV and emetine. The antiviral activity was evaluated by luciferase assay. Data represent mean values ± the SE of triplicate determinations from a representative of three independent experiments.

Screening of the LOPAC library using a pp28-luciferase HCMV Towne identified emetine as a potential HCMV inhibitor. A dose response curve was generated to confirm the anti-HCMV activity of emetine. The EC_50_ of emetine against pp28-luciferase Towne was 40±1.72 nM, and the CC_50_ in non-infected human foreskin fibroblasts (HFFs)—8±0.56 μM, yielding a selectivity index of 200. The Hill slope of the concentration-response curve was 3.1, indicating a robust virus inhibition at higher concentrations [17] (Fig 1A and 1B). A ganciclovir (GCV)-resistant pp28-luciferase Towne was also inhibited by emetine. Inhibition of HCMV and mouse CMV (MCMV) by emetine was confirmed by plaque reduction assay (S1 Table). The activity of emetine against herpesvirus 1 (HSV-1) and HSV-2 was determined by luciferase and plaque assay in HFFs, respectively, revealing virus inhibition at nM concentrations (S1 Table). The expression of HCMV proteins IE1/2, UL44 and pp65 was significantly reduced by emetine at 72 hours post infection (hpi) (Fig 1C). Combination of emetine and GCV was synergistic in HCMV inhibition, as determined by the Bliss model (Fig 1D). These results indicate robust *in vitro* inhibition of HCMV, GCV-resistant HCMV, MCMV and HSVs at nM concentrations of emetine. At these concentrations emetine did not inhibit protein synthesis in non-infected or HCMV-infected cells (S1 Fig), in agreement with previous studies [18,19].

### Emetine inhibits HCMV replication after entry but before initiation of DNA replication

**Fig 2:**
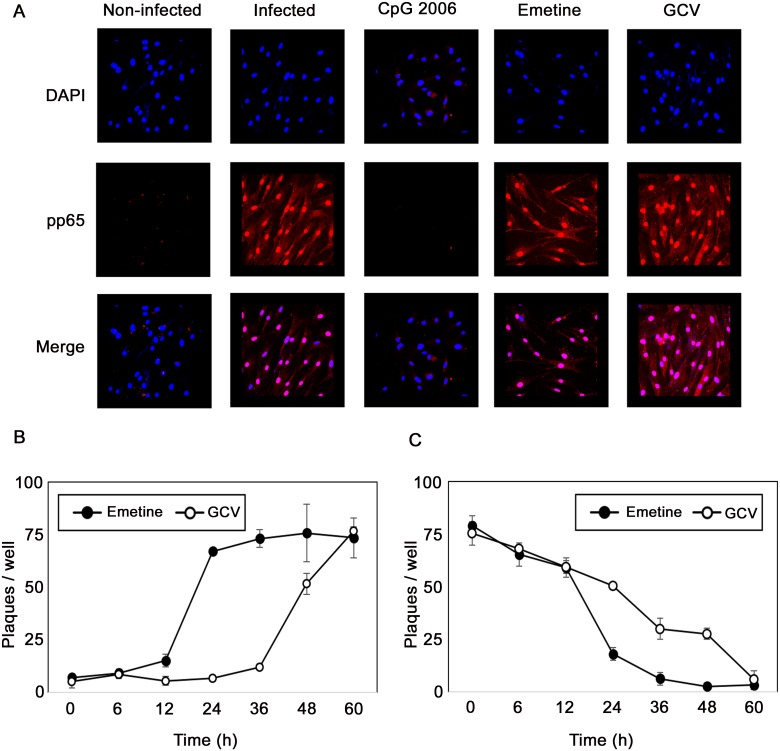
Emetine is an early inhibitor of HCMV replication. **A)** Emetine does not inhibit HCMV entry. Cells were treated with emetine (75 nM), GCV (10 μM), and CpG 2006 (10 μM) 24 h prior to infection. Cells were infected with HCMV and treated with compounds for 90 min. Immunofluorescence staining was performed with mouse monoclonal anti-pp65 antibody. The fluorescence of rhodamine anti-mouse IgG and DAPI was visualized and merged using a Nikon Eclipse E-800 fluorescence microscope. **B)** Emetine has an early activity against HCMV. Cells were infected with HCMV Towne, and compounds were added at 0, 6, 12, 24, 36, and 48 hpi (Add on). Culture supernatants (10%) were collected at 72 hpi for a plaque assay after 14 days. **C)** Cells were infected with HCMV Towne and treated with compounds immediately after virus adsorption. Compounds were removed at 0, 6, 12, 24, 36, and 48 hpi (Removal). Culture supernatants were collected at 72 hpi for titration by plaque assay. Data represent mean ± SE of triplicate determinations from a representative of two independent experiments.

Using immunofluorescence assay for pp65, neither emetine nor GCV inhibited viral entry, but CPG 2006 (a TLR9 ligand), used as positive control, did (Fig 2A). In add-on and removal assays emetine or GCV were added or removed at 0, 6, 12, 24, 36, 48 and 60 hpi, and supernatants were collected at 72 hpi for titration of infectious virus by plaque assay. Addition of emetine after 12 h resulted in its loss of activity against HCMV (Fig 2B, *p*< 0.0001). The removal assay revealed that emetine was required for at least 24 h to fully inhibit HCMV (Fig 2C, *p*< 0.05)). Thus, HCMV inhibition occurred during the immediate-early to early stages of HCMV replication.

### Pharmacokinetics (PK) and efficacy of emetine against mouse CMV

**Fig 3:**
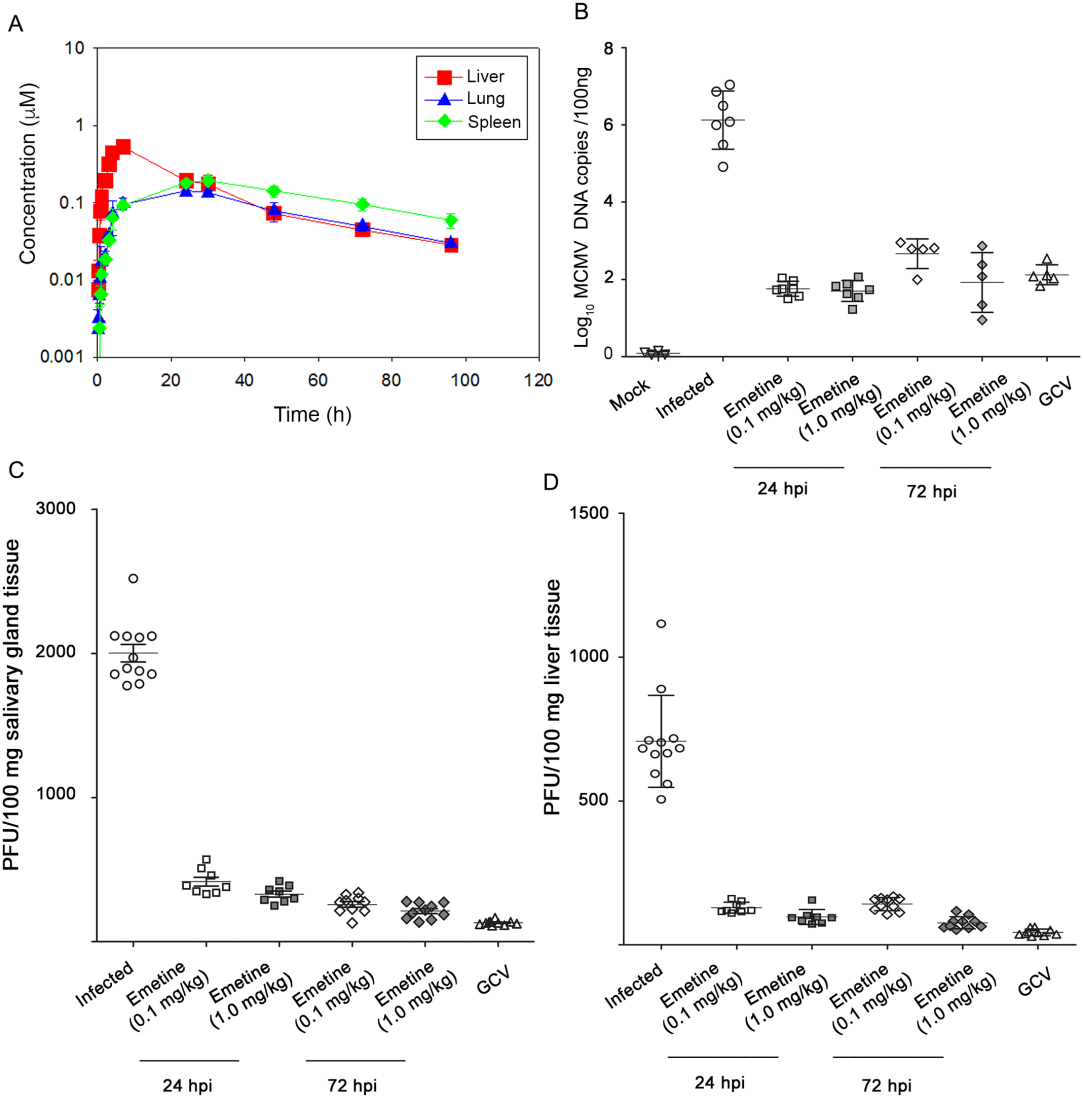
Emetine achieves high tissue concentrations and is efficacious against MCMV replication. **A)** *In vivo* pharmacokinetics of emetine. Plasma, liver, lung and spleen samples were collected at the indicated time points after single oral administration of emetine at 0.1 mg/kg in male BALB/c mice. Concentrations were measured using UPLC-MS/MS methods. For plasma, the concentrations observed were mostly below the level limit of quantification. **B)** Quantitative real-time PCR of viral gB was performed on DNA extracted from blood at day 14 post infection. Plaque assay was performed from salivary glands **C)** or liver **D)** collected at day 14 post infection. Emetine was administered orally starting 24 hpi or 72 hpi at 0.1 or 1.0 mg/kg every 3 days. GCV dose was 10 mg/kg/dose administered intraperitoneally twice daily.

Since PK data with low dose emetine have not been reported before, we conducted a PK study in BALB/c mice after single oral administration of 0.1 mg/kg emetine. *In vivo* exposures of emetine in plasma, liver, lung and spleen were monitored (S2 Table). Emetine achieved levels that exceeded its *in vitro* EC_50_ against HCMV (Fig 3A) and its calculated half-life was 35 h.

**Fig 4:**
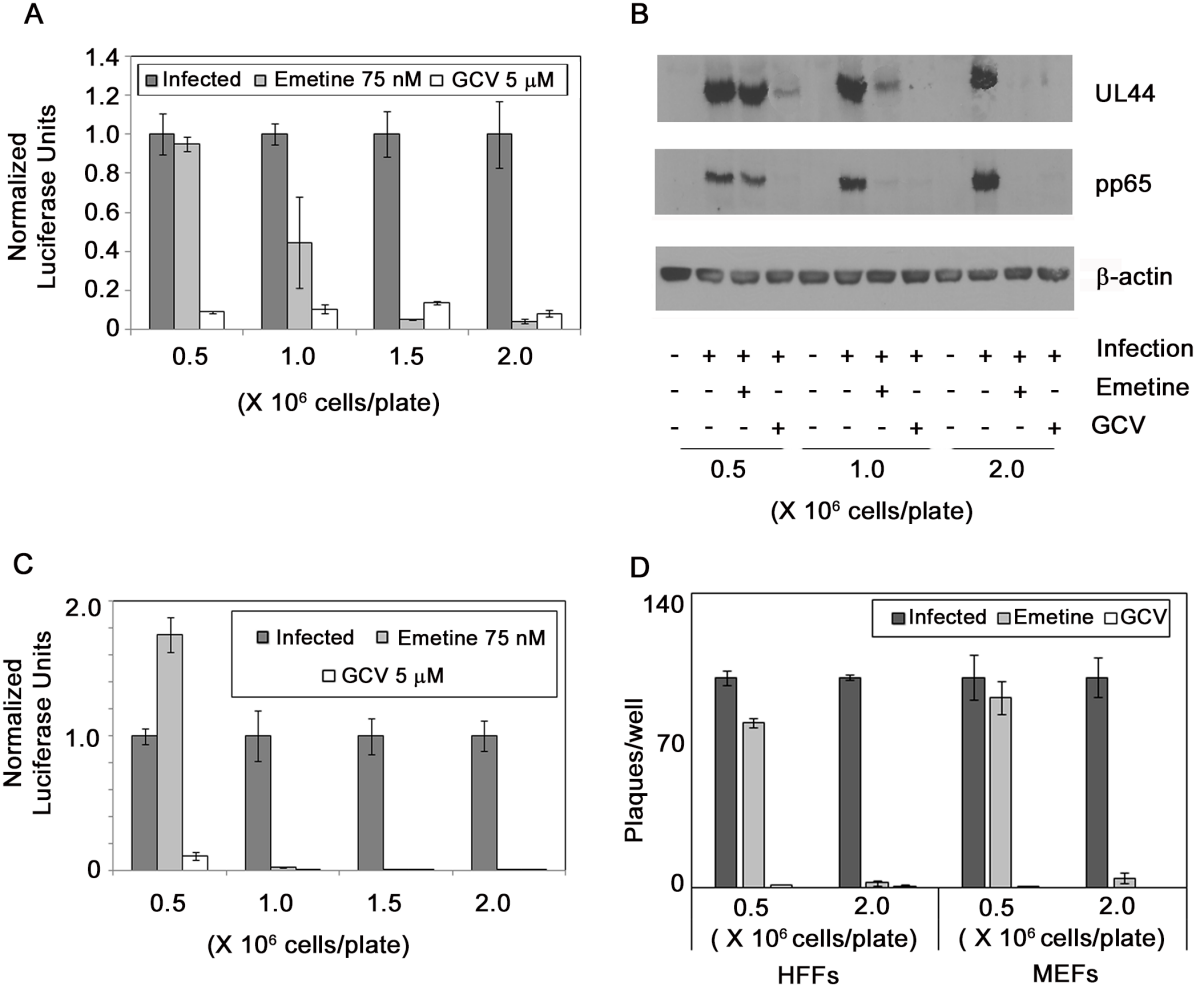
The anti-HCMV activity of emetine is reduced in low-density HFFs. **A)** Cells were seeded at indicated densities in a 96-well plate, infected with pp28-luciferase HCMV Towne (MOI = 1) and treated with emetine (75 nM) or GCV (5 μM). Luciferase activity was measured at 72 hpi and normalized to the activity of infected untreated cells. Data represent mean ± SE of triplicate determinations from a representative of two independent experiments. **B)** Same cell lysates as in A were collected for Western blotting at 72 hpi and expression of viral proteins UL44 and pp65 was determined. **C)** Supernatants from each well as in A were used to infect cells (1 million cells in a 96-well plate) in the corresponding well and luciferase activity was measured at 72 hpi. **D)** Cells were seeded into 12-well plates at 0.5 and 2 million cells/plate, infected with 100 PFU/well of HCMV Towne or MCMV, and treated with emetine (75 nM) or GCV (5 μM). After 10 days (for HCMV) or 3 days (for MCMV) plaques were stained with crystal violet and the number of plaques enumerated. Data shown are average of 2 wells (±SD) for a representative experiment from two different experiments.

**Fig 5:**
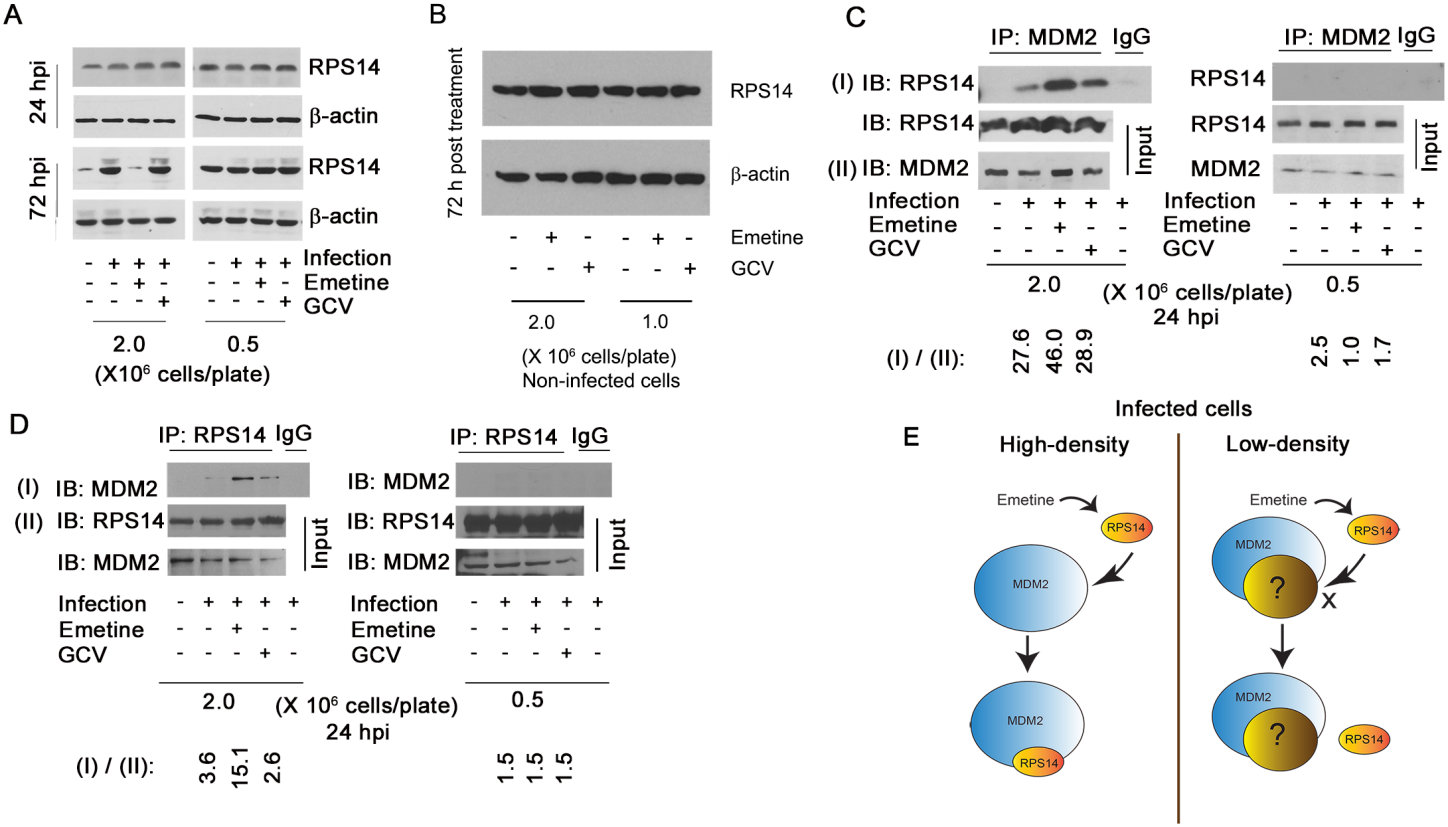
Emetine induces an interaction between MDM2 and RPS14 in infected high-density cells. **A)** Emetine abrogates infection-mediated overexpression of RPS14 at 72 hpi. Cells were seeded at 0.5 or 2 million cells/plate, infected with Towne HCMV and treated with emetine (75 nM) or GCV (5μM). Lysates were collected at 24 or 72 hpi for Western blotting. **B)** HFFs were seeded at 1 or 2 million cells/plate and treated with emetine or GCV. Lysates were collected at 72 hpi for Western blotting. **C)** Cells were seeded at 0.5 or 2 million/plate in 100 mm dishes, infected with Towne HCMV followed by treatment with emetine (75 nM) or GCV (5μM) for 24h. MG132 (10 μM) was added after 12 h. At 24 hpi, lysates were collected and subjected to IP with anti-MDM2 followed by immunoblotting with anti-RPS14 antibody or **D)** In reverse reaction, IP was performed with anti-RPS14 followed by immunoblotting with anti-MDM2 antibody. Densitometry analysis was performed to normalize immunoprecipitated protein to its input level. **E**) A model showing the interaction of MDM2 and RPS14 in high-density but not in low-density cells.

**Fig 6:**
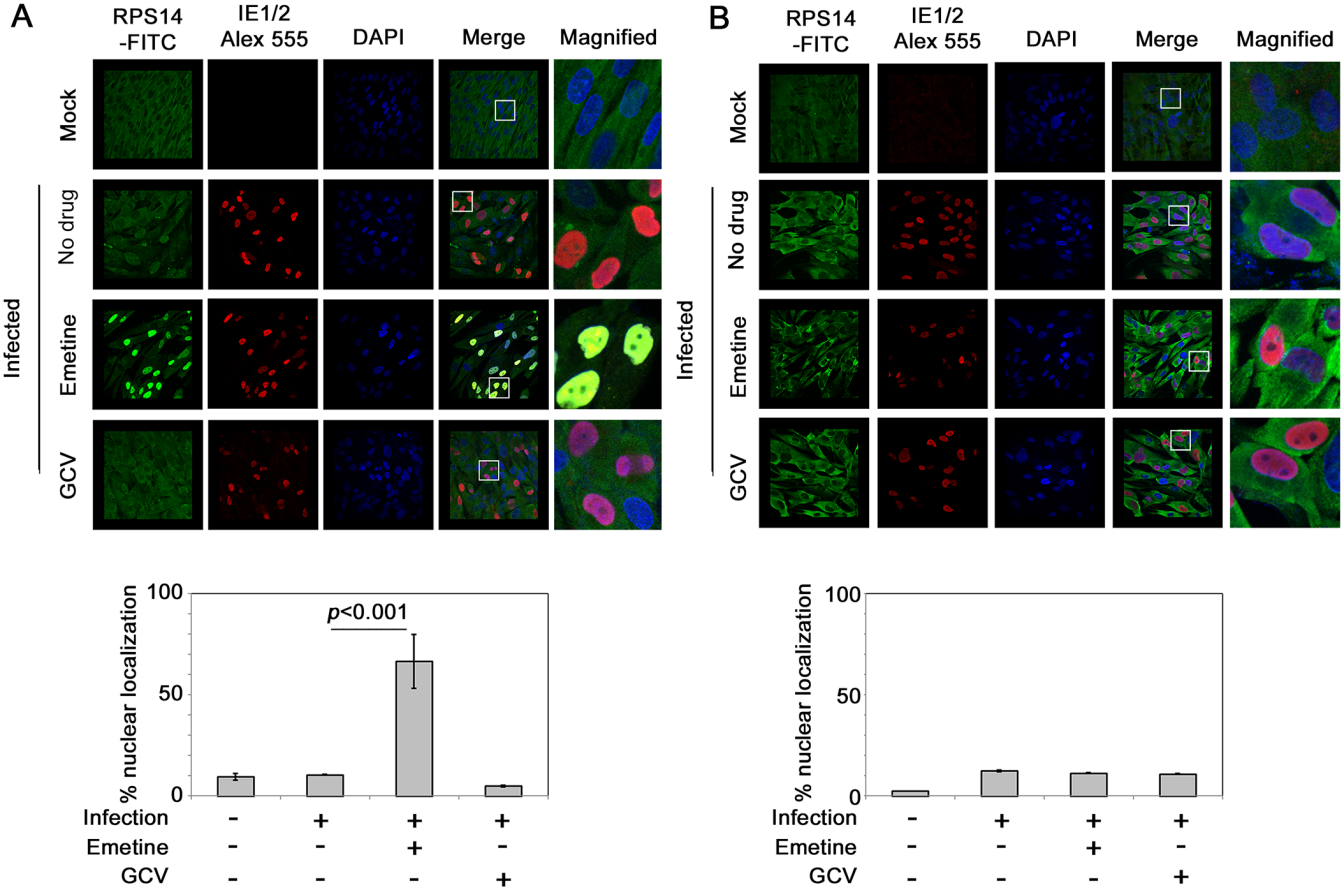
Emetine induces nuclear translocation of RPS14 in infected high-density cells at 24 hpi followed by cytoplasmic relocalization. **A)** Cells were seeded at 2 million/4-well chamber slide, infected and treated with either emetine (75 nM) or GCV (5μM) for 24 h. Cells were stained with IE1/2 (Alexa 555:Red) for evidence of infection and RPS14 (FITC: Green) and nuclear DAPI. Stained slides were subjected to confocal microscopy and colocalization was studied and quantified using NIS elements software (Nikon). **B)** The same procedure was repeated at 72 hpi. The images shown are a representative of two independent experiments. Percent nuclear localization is represented as Mean ± SD from two different fields of at least 40 cells per condition.

The effect of emetine on MCMV replication was tested. BALB/c mice (3–4 week old) were infected intraperitoneally with tissue-culture derived MCMV [10^6^ plaque forming units (PFU)/mice] and treated with 0.1 or 1 mg/kg of emetine orally every three days beginning at 24 h or 72 h after infection until day 11 post infection. On day 14 post infection, mice were euthanized, intracardiac blood was collected and tissues were harvested and assayed for MCMV replication by plaque assay. Emetine treatment resulted in 2 to 4 log10 decrease in MCMV DNA copy number in blood, compared to infected control (Fig 3B, *p*<0.001). All treatment regimens resulted in 4–6 fold reduction of viral PFUs in salivary gland (Fig 3C) and 3–6 fold reduction in liver (Fig 3D, *p*<0.0001). Both doses of emetine were highly efficacious in MCMV inhibition, when administered at 24 or at 72 hpi.

### HCMV inhibition by emetine depends on cell confluency at the time of infection

The following *in vitro* studies were undertaken to elucidate the mechanism of anti-HCMV activity of emetine and should not point to *in vivo* efficacy, since MCMV was inhibited with emetine, and most HCMV-infected cells *in vivo* are high-density. We observed that cell density at the time of infection determined HCMV inhibition by emetine. Lack of HCMV inhibition by emetine was not because of selection of resistant viruses. Increasing emetine concentration did not select for resistant mutants, while GCV selected for a C607Y mutation in UL97, confirmed by sequencing. To investigate the contribution of cell density to the anti-HCMV activities of emetine HFFs were seeded at 0.5–2 million cells/plate followed by infection and drug treatment. HCMV inhibition by emetine improved as cell confluence at the time of infection increased. There was no reduction in normalized pp28-luciferase activity by emetine at low cell density, whereas with increased cell density significant reduction was observed (Fig 4A). Western blots revealed decreased expression of viral UL44 and pp65 with emetine at high but not at a lower cell density (Fig 4B). Second cycle infection similarly showed dependence of HCMV inhibition by emetine on cell density; there was 1.5-fold increase in pp28-luciferase activity at 0.5 million cells/plate (*p*>0.5), and near complete inhibition at 1 and 2 million cells/plate (*p*<0.001), compared to infected control (Fig 4C). Plaque reduction assays showed 30-fold reduction in the number and size of plaques with emetine at 2 million cells/plate (*p*<0.001) as compared to 0.5 million cells/plate (Fig 4D, *p*>0.05). A plaque reduction assay of MCMV similarly showed a cell-density dependent response to emetine (Fig 4D, *p*<0.001). GCV inhibited HCMV and MCMV irrespective of cell density (*p*<0.001). These results suggest that cell cycle related activities may determine HCMV inhibition by emetine.

### Emetine induces an interaction between RPS14 and MDM2 in HCMV-infected cells

Resistance to emetine in Chinese hamster ovary cells was associated with mutations in the ribosomal protein S14 (RPS14) [20]. This protein was reported to interact with MDM2-p53, major regulators of cell cycle progression [21]. We theorized that the regulation of RPS14 by emetine determines its anti-HCMV activities in high-density cells. RPS14 expression was measured in high-density and low-density cells, infected or non-infected. In high-density cells, RPS14 expression was induced at 72 hpi, and reduced with emetine (Fig 5A, left). However, in infected low-density cells RPS14 expression was unchanged (Fig 5A, right). At 24 hpi the expression level of RPS14 was unchanged in high-density or in low-density cells (Fig 5A, upper panel, left and right). Irrespective of cell density, there was no change in RPS14 expression in non-infected cells treated with emetine (Fig 5B). Thus, induction of RPS14 expression at 72 hpi and its reduction by emetine was specific to infected high-density cells. The anti-HCMV activity of emetine occurred at the immediate early-early stage of HCMV replication (Fig 2A and 2B), thus, we suspected that the changes in RPS14 expression at 72 hpi represent an outcome of an earlier event.

Since RPS14 was reported to interact with MDM2 during ribosomal stress [21], the outcome of the MDM2-RPS14 interaction during HCMV infection and emetine treatment was investigated. As an E3 ubiquitin ligase, MDM2 degrades proteins to which it binds [22], therefore experiments were performed in the presence of the proteasomal inhibitor, MG132. At 24 hpi emetine strongly induced the interaction between RPS14 and MDM2 in high-density cells, but not in low-density cells (Fig 5C). Similarly, a reverse immunoprecipitation (IP) showed enhanced interaction between RPS14-MDM2 in high-density cells (Fig 5D). Anti-RPS19 and isotype control antibodies further confirmed the specificity of the RPS14-MDM2 interaction (S2A Fig). Emetine did not induce an interaction between RPS19 and MDM2. Since the inhibition of MCMV replication by emetine was also cell-density dependent *in vitro* (Fig 4D), the RPS14-MDM2 interaction was tested in MCMV-infected mouse embryonic fibroblasts (MEFs, S3A Fig), again revealing RPS14-MDM2 interaction only in high-density MEFs. In non-infected HFFs emetine did not induce an interaction between RPS14 and MDM2 (S4A Fig). A summary model shows the differences in RPS14 and MDM2 interaction between infected high-density and low-density cells with emetine treatment (Fig 5E). Since RPS14 expression was reduced at 72 hpi with emetine treatment (Fig 5A, left), the enhanced interaction between MDM2 and RPS14 at 24 hpi suggested that MDM2 may be targeting RPS14 for degradation in infected cells.

### Emetine induces nuclear translocation of RPS14 in infected high-density cells and targets it for ubiquitination and degradation

**Fig 7:**
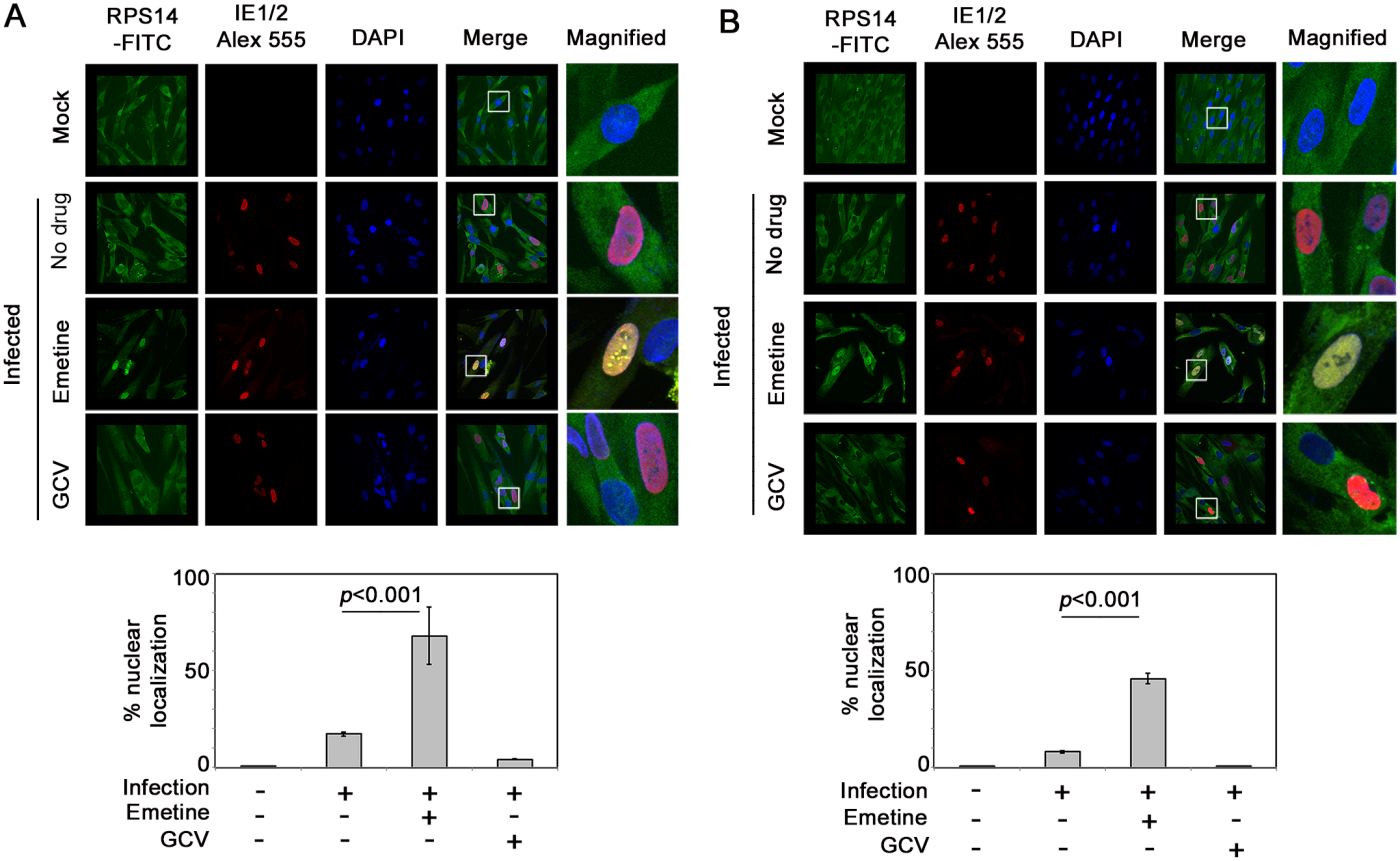
Nuclear localization of RPS14 persists during infection and emetine treatment in low-density cells. **A)** Cells were seeded at 0.5 million/4-well chamber slide, infected and treated with either emetine (75 nM) or GCV (5μM) for 24 h. Cells were stained with IE1/2 (Alexa 555:Red) for evidence of infection and RPS14 (FITC: Green) and nuclear DAPI. Stained slides were subjected to confocal microscopy and nuclear localization was quantified using NIS elements software (Nikon). **B)** The same procedure was repeated at 72 hpi. The images shown are a representative of two independent experiments. Percent nuclear localization is represented as mean ± SD of at least 25 cells per condition from two different fields in the slide.

**Fig 8:**
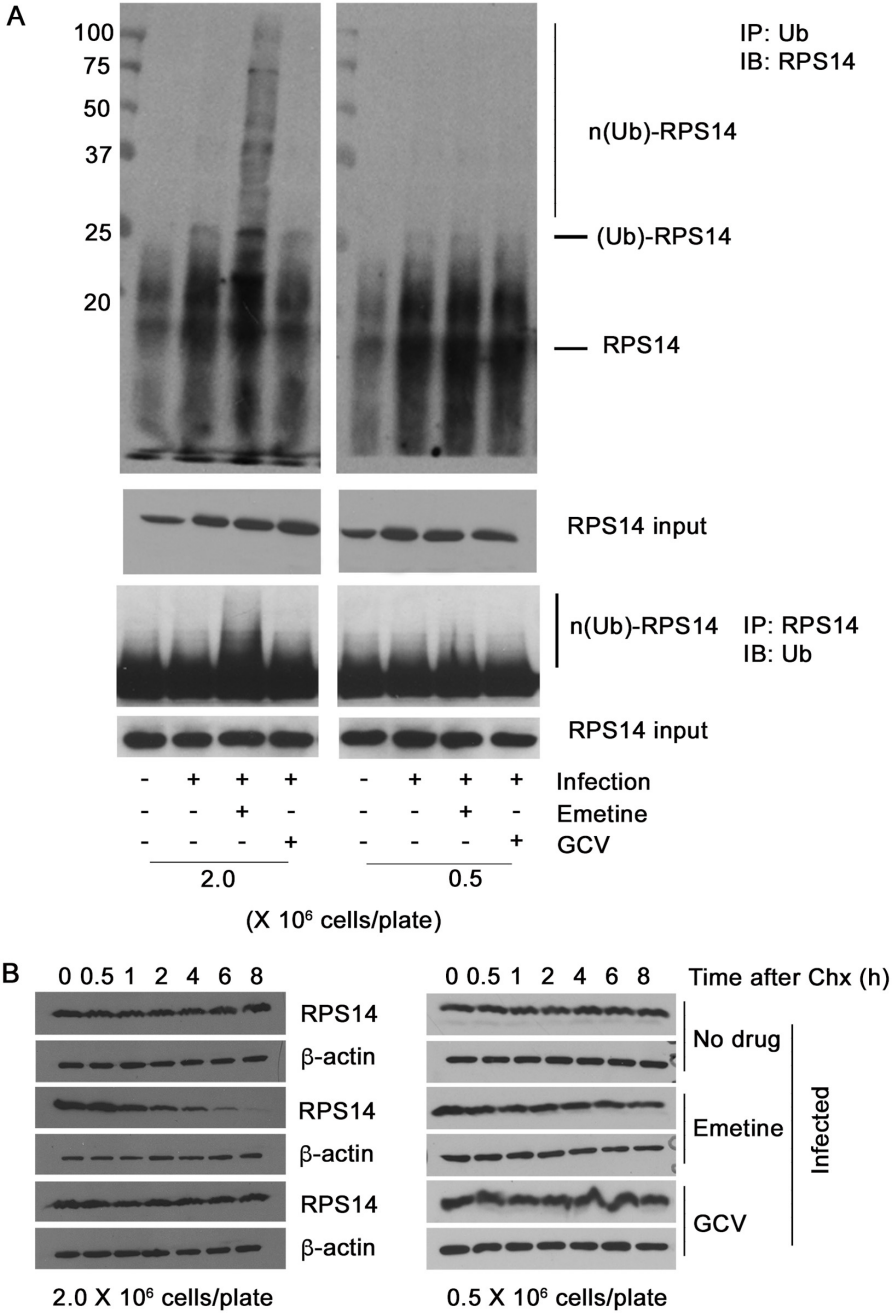
RPS14 is ubiquitinated and degraded in infected high-density cells. **A)** Cells were seeded at 0.5 or 2 million/plate in 100 mm dishes, infected with HCMV Towne followed by treatment with emetine (75 nM) or GCV (5μM) for 72h. MG132 (10 μM) was added 12 h before harvest. Lysates were collected at 72 hpi and subjected to IP with anti-Ubiquitin antibody followed by immunoblotting with anti-RPS14 antibody or IP with anti-RPS14 antibody followed by immunoblotting with anti-Ubiquitin antibody. The mono- and poly- ubiquitinated forms of RPS14 are depicted as (Ub)-RPS14 and n(Ub)-RPS14, respectively. **B)** HCMV-infected cells (left: high-density right: low-density) were treated with emetine or GCV. At 24 hpi cycloheximide (CHX, 100 μg/mL) was added. Cells were harvested at the indicated time intervals up to 8 h following CHX for SDS-PAGE analysis.

To interact with MDM2, we predicted that RPS14 would translocate into the nucleus. Using confocal microscopy, RPS14 was located in the cytoplasm of non-infected or HCMV-infected cells (Fig 6, upper two panels). In high-density cells emetine treatment induced RPS14 translocation into the nucleus at 24 hpi (Fig 6A, 66% localization, *p*<0.001), but at 72 hpi nuclear localization of RPS14 decreased to a similar level as in non-treated cells (Fig 6B, 11% localization, *p*>0.5). However, in infected low-density cells nuclear localization of RPS14 was observed at both 24 and 72 hpi (Fig 7A & 7B, *p*<0.001), indicating that although emetine could initially induce nuclear translocation of RPS14 in infected low-density cells, subsequent localization changes did not occur, likely because RPS14 did not interact with MDM2. Since in emetine treated high-density cells, RPS14 expression and its nuclear localization were decreased at 72 hpi (Figs 5A and 6B), while a strong interaction with MDM2 was observed at 24 hpi (Fig 5C and 5D) we conjectured that RPS14 may be targeted for degradation. MG132-treated samples were pulled-down with anti-Ubiquitin antibody and detected with anti-RPS14 antibody. In both cell densities, RPS14 ubiquitination mildly increased with infection. However, only in high-density cells, emetine increased both the mono and poly-ubiquitinated forms of RPS14 (Fig 8A, left). A reverse IP similarly showed increased RPS14 ubiquitination in high-density cells. In agreement with RPS14 ubiquitination, at 72 hpi there was almost no nuclear RPS14 in high-density infected emetine treated cells, indicating that emetine targets RPS14 for ubiquitination and degradation (Fig 6B). In infected low-density cells RPS14 ubiquitination was not increased with emetine, in agreement with its persistent nuclear localization at 72 hpi (Fig 8A, right). The rate of degradation of RPS14 in high- and low-density cells was tested with the protein synthesis inhibitor, cycloheximide. As expected, RPS14 showed significant degradation in infected high-density cells, compared to infected only or infected GCV-treated cells (Fig 8B, left). However, there was no substantial degradation of RPS14 in the low-density cells under any of the conditions used (Fig 8B, right). Taken together, emetine treatment of infected high-density cells results in early RPS14 translocation into the nucleus of infected cells followed by its relocalization into the cytoplasm for ubiquitination and degradation, ultimately resulting in decreased RPS14 expression at 72 hpi (Fig 5A, left).

### Emetine disrupts HCMV-mediated MDM2-p53 interaction

**Fig 9:**
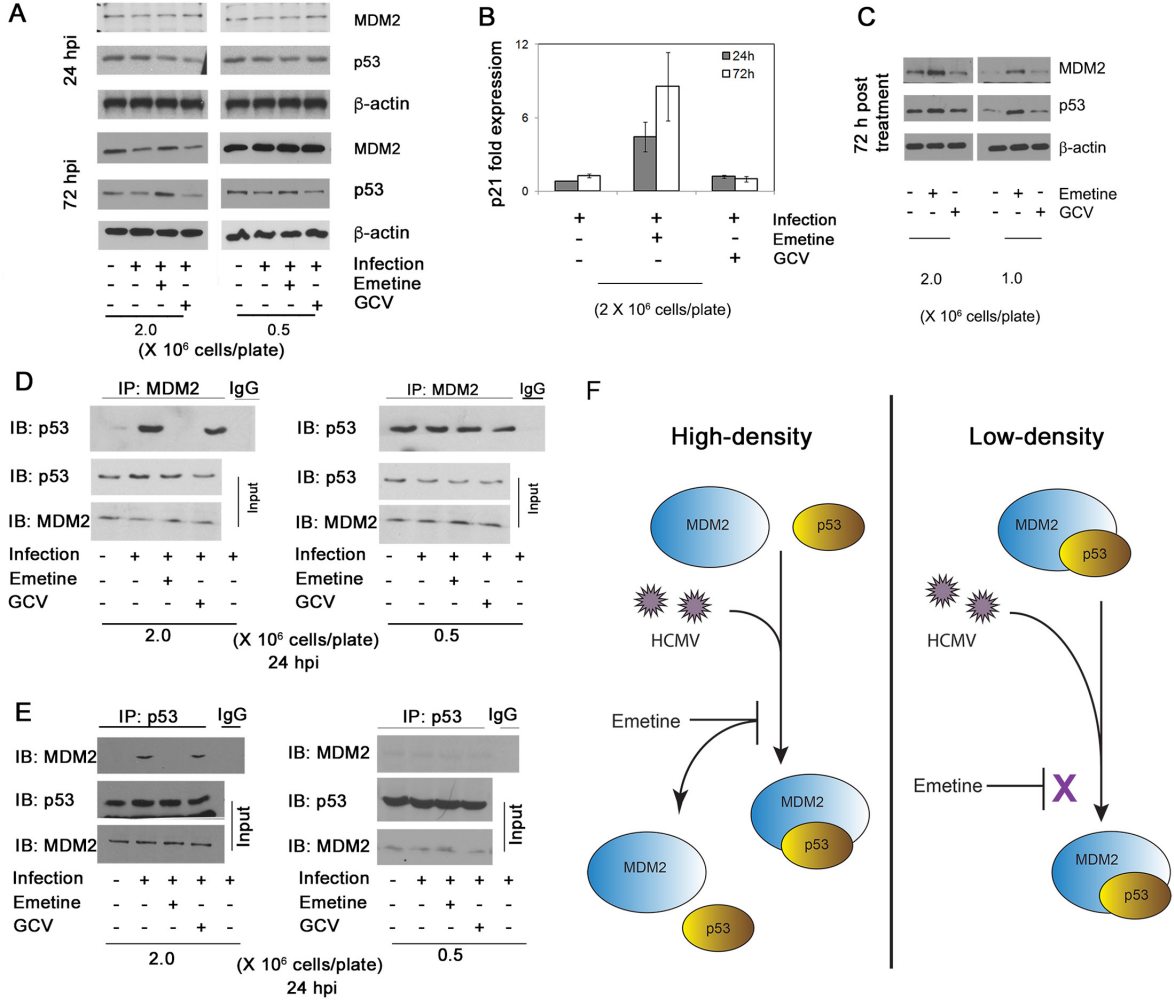
Emetine disrupts HCMV mediated MDM2-p53 complex. **A)** MDM2 and p53 expression increases with emetine treatment. Cells were seeded at 0.5 or 2 million cells/plate, infected with HCMV Towne followed by treatment with emetine (75 nM) or GCV (5 μM). Cell lysates were harvested at 24 or 72 hpi for Western blotting. **B)** Cells were seeded at 2 million cells/plate, infected with HCMV Towne and treated with emetine (75 nM) or GCV (5 μM). RNA was harvested and qRT-PCR was performed for p21. Data represent mean ±SE of triplicate determinations from a representative of three independent experiments **C)** HFFs were seeded at 1 or 2 million cells/plate and treated with emetine (75 nM) or GCV (5 μM). Cell lysates were harvested at 72 hpi for Western blotting. **D)** HFFs were seeded at either 0.5 or 2 million/plate in 100 mm dishes, infected with HCMV Towne followed by treatment with emetine (75 nM) or GCV (5μM) for 24h. MG132 (10 μM) was added after 12 h. At 24 hpi, lysates were collected and subjected to IP with anti-MDM2 antibody followed by immunoblotting with anti-p53 antibody or **E)** IP with anti-p53 antibody followed by immunoblotting with anti-MDM2 antibody. **F**) A model depicting the mechanism by which emetine disrupts HCMV mediated MDM2-p53 interaction in high-density cells, but not in cycling low-density cells.

Since emetine inhibited HCMV in high-density, but not in low-density cells, and induced an interaction between MDM2 and RPS14 in the former, the expression level of p53 and MDM2 was measured in the different cell densities at 24 and 72 hpi. MDM2 expression was reduced after infection and increased with emetine treatment at 72 hpi in high-density cells (Fig 9A). The expression of p53 was unchanged with infection and increased with emetine treatment at 72 hpi (Fig 9A). There was no difference in the expression of either protein among the different conditions at 24 hpi or in infected low-density cells at 72 hpi (Fig 9A). The downstream activity of p53 was measured by quantitative reverse transcriptase (qRT)-PCR for p21 (22). Emetine treatment resulted in 5.4- and 6.7- fold increase in p21 mRNA at 24 and 72 hpi, respectively in high-density cells (Fig 9B, *p*<0.01). In non-infected cells, the expression of p53 and MDM2 was increased with emetine, indicating both proteins were stabilized with emetine, irrespective of infection (Fig 9C).

RPS14 was reported to bind to the acidic domain of MDM2, which is also the binding site for p53 [23]. Therefore, the effect of infection and emetine treatment on MDM2-p53 interaction was studied. Since p53 is degraded by the E3 ubiquitin ligase activity of MDM2 [24,25], MG132 treated samples were used for IP. The interaction between MDM2 and p53 was favored upon infection, disrupted with emetine in high-density cells, but not in low-density cells (Fig 9D). A reverse IP confirmed this interaction in infected cells and loss thereof with emetine (Fig 9E), and isotype control antibodies did not pull down either MDM2 or p53 (S2B Fig), indicating that emetine disrupts HCMV-induced interaction of MDM2-p53 (model, Fig 9F). Similar interactions were observed in MCMV-infected MEFs (S3B Fig). Since the expression of p53 and MDM2 was increased with emetine in non-infected cells, MDM2-RPS14 interaction was investigated in non-infected cells. Emetine could not induce MDM2-RPS14 interaction in non-infected cells (S4A Fig). Therefore, emetine could stabilize MDM2 and p53 irrespective of infection but its ability to associate RPS14 with MDM2 resulting in p53 activation was achieved only in infected high-density cells, probably because in non-infected cells it could not trigger RPS14 localization into the nucleus (S4B Fig).

Finally, the enhanced interaction between RPS14-MDM2 could have effects on HCMV proteins that bind to MDM2. The immediate early 2 (IE2) was reported to interact with MDM2 [26], therefore the effect of emetine on IE2-MDM2 interaction was tested in HEK293 cells. An IP was performed after IE1/2 transfection and emetine treatment. In emetine-treated cells, the interaction between MDM2 and IE2 was significantly decreased, while GCV had no effect on this interaction (S5 Fig). These results suggest that emetine-induced occupancy of MDM2 by RPS14 may prevent it from binding IE2.

### RPS14 is indispensable for HCMV inhibition by emetine

**Fig 10:**
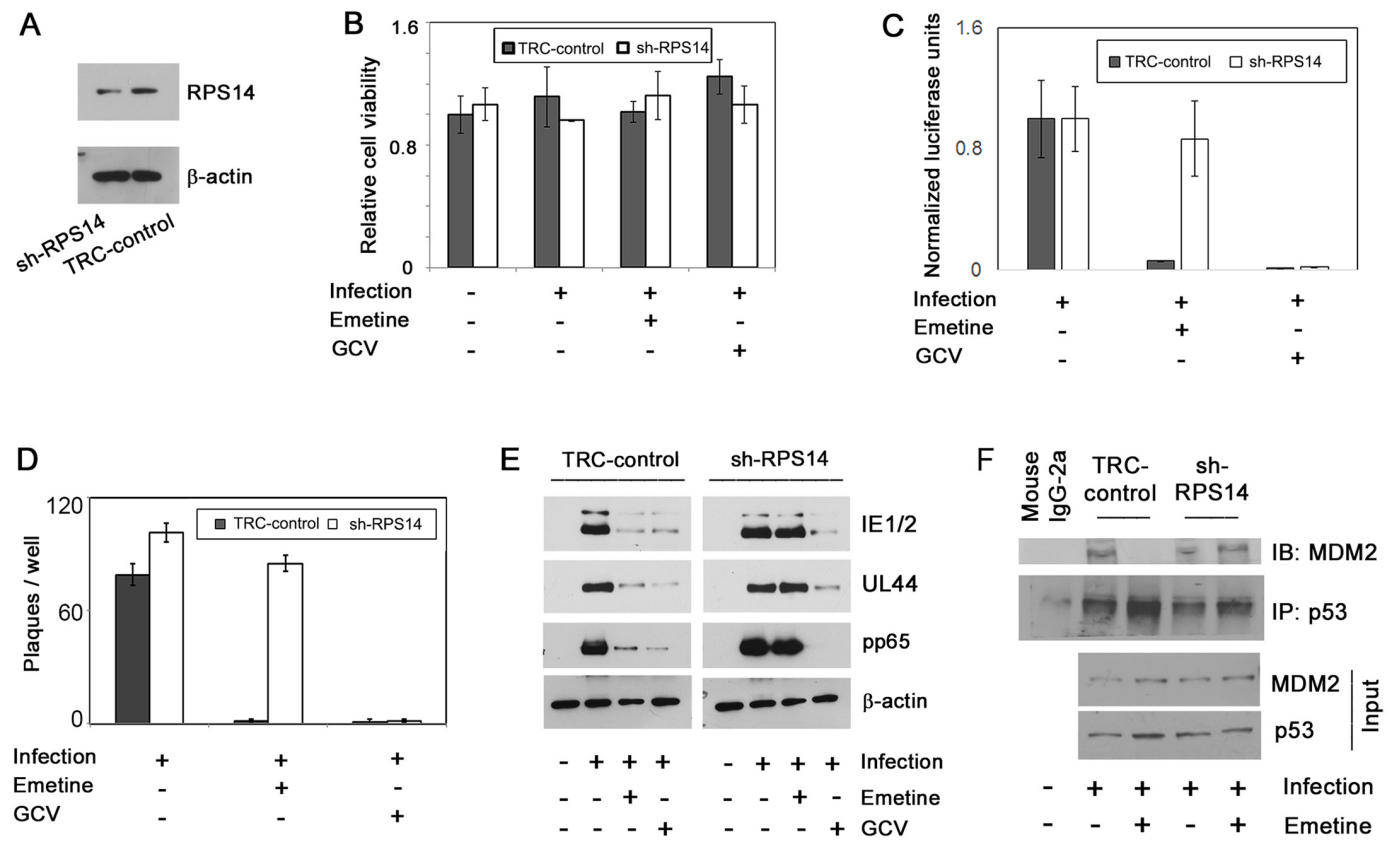
Emetine loses its anti-HCMV activity in RPS14 knockdown cells. **A)** RPS14 knockdown HFFs (sh-RPS14) were generated from lentiviral system and expression of RPS14 in the knockdown cells was compared to TRC control cells by Western blotting. **B)** sh-RPS14 and TRC control cells were seeded at 2 million/plate, infected and treated with emetine (75 nM) or GCV (5μM) for 72h. Cell viability was determined after 72 h. **C)** Luciferase activity was measured in cell lysates. **D)** sh-RPS14 and TRC control cells were seeded at 2 million/plate, in a 12-well plate and infected 24 hours later at 100 PFU/well. After 90 min virus was aspirated, and DMEM containing 4% fetal bovine serum (FBS) with 0.5% carboxymethyl-cellulose, were added with the compounds at indicated concentrations into triplicate wells. Plaques were counted after incubation at 37°C for 8 days. **E)** Same cell lysates as in B were used to determine HCMV pp65, UL44 and IE1/2 expression. **F)** sh-RPS14 and TRC-control cells were infected and treated with emetine (75 nM) for 24h. MG132 (10 μM) was added after 12 h. At 24 hpi, lysates were collected and subjected to IP with anti-p53 antibody followed by immunoblotting with anti-MDM2 antibody.

As emetine induced nuclear translocation of RPS14 and its interaction with MDM2, we investigated the anti-HCMV activity of emetine in RPS14 knockdown cells (sh-RPS14). The expression of RPS14 was reduced in sh-RPS14 cells as compared to its expression in TRC-control shRNA cells (Fig 10A). Cell viability was similar between control transduced and sh-RPS14 cells during infection and drug treatment (Fig 10B). In high-density sh-RPS14 cells, emetine was unable to inhibit HCMV, evident from luciferase assay at 72 hpi (Fig 10C), a plaque reduction assay, (Fig 10D) and expression of pp65, UL44 and IE1/2 at 72 hpi (Fig 10E), indicating the requirement of RPS14 for emetine activities. In shRPS14 cells a stable interaction between MDM2 and p53 was observed despite emetine treatment, while as expected the interaction was disrupted in TRC control cells (Fig 10F). These results indicate that a certain level of RPS14 is required for its interaction with MDM2, in emetine treated HCMV-infected cells, resulting in displacement of IE2 and p53 from MDM2.

## Discussion

The existing antiviral drugs effectively suppress HCMV replication, but their considerable side effects, and selection of resistant viral strains, call for the identification of new HCMV inhibitors [27]. HCMV perturbs a myriad of cellular signaling pathways for its own benefit of replication and survival [28], some of which could serve as novel targets for virus inhibition. We report here on the anti-HCMV activities of emetine at low nM concentration (Fig 1), and its mode of action through modifying the interaction of MDM2-RPS14/-p53, thus providing a novel host-dependent antiviral approach. Emetine inhibited GCV-resistant HCMV, MCMV and HSV1&2 at nM concentrations as well (S1 Table). Inhibition of HCMV replication by emetine occurred after virus entry and before GCV activities (Fig 2). MCMV was inhibited at low 0.1 mg/kg. There was no significant difference in MCMV inhibition between 0.1 and 1 mg/kg. Plaque number was lower in the liver compared to the salivary gland (as expected), but virus was inhibited in both organs (Fig 3) [29].

**Fig 11:**
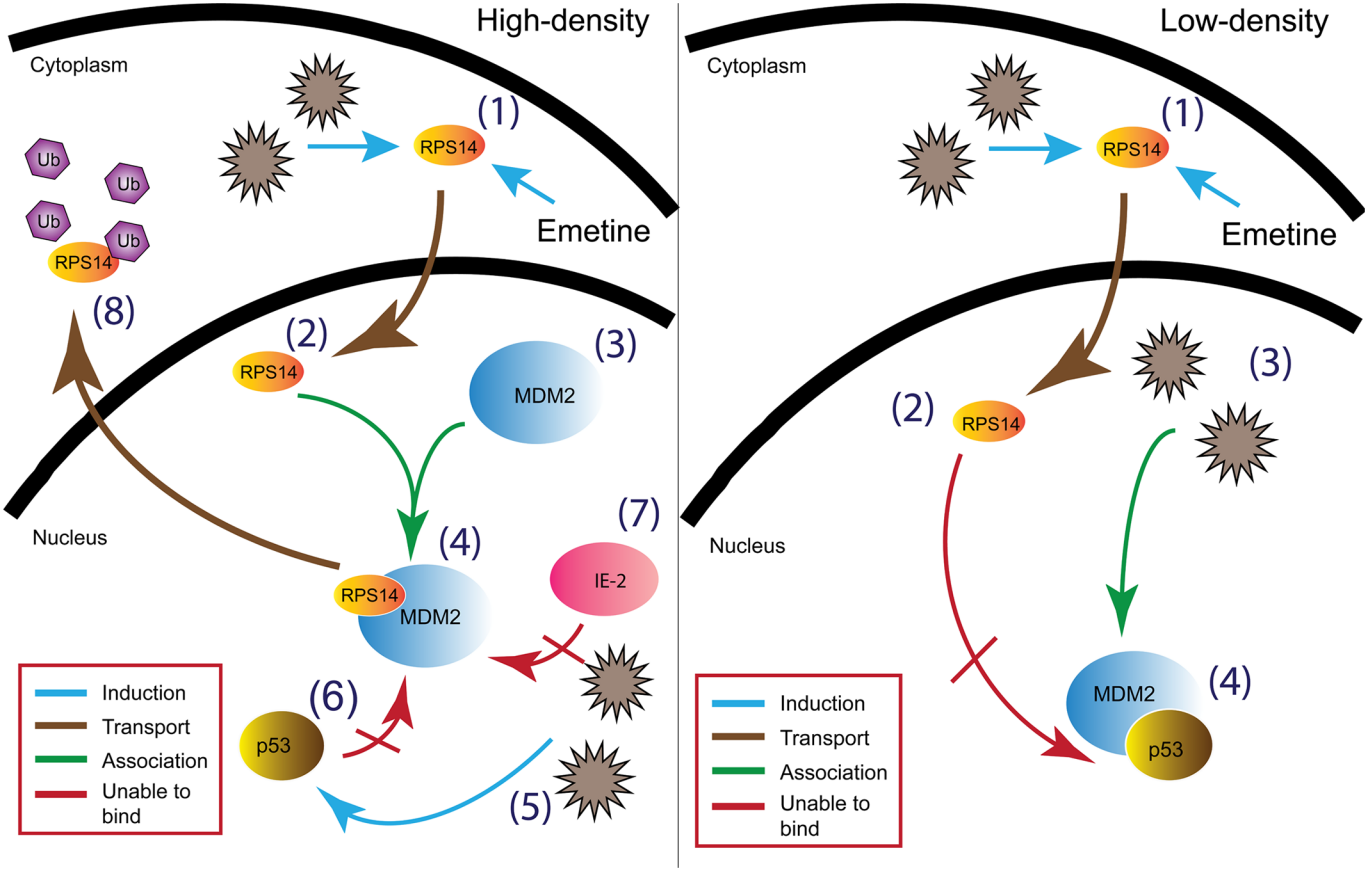
Model of HCMV inhibition by emetine. In high-density infected cells (A) emetine induces (1) nuclear translocation of RPS14 (2) followed by RPS14 binding to MDM2 (3 & 4) resulting in disruption of the interaction between MDM2-p53 (6) and MDM2- viral IE2 (5 & 7), and by RPS14 ubiquitination and degradation (8). In low-density infected cells (B) although emetine induces (1) nuclear translocation of RPS14 (2), it is unable to interact with MDM2 (4) which is already bound to p53 to facilitate virus replication (3).

The observed lack of activity of emetine against HCMV and MCMV in low-density as compared to high-density cells (Fig 4), and a previous study that correlated emetine resistance in Chinese hamster ovary cells with mutations in RPS14 [30], prompted us to investigate the functional role of RPS14 in HCMV infection and emetine treatment (summary model, Fig 11). HCMV induces multiple ribosomal proteins, but whether any of these ribosomal proteins may be utilized by drugs to inhibit virus replication has not been studied [31]. Our results show that HCMV induces RPS14, likely as a strategy for viral protein synthesis (Fig 5A). In low-density non-infected cells, higher expression of RPS14 was observed compared to high-density cells, suggesting the former are more active in synthesizing proteins for ongoing growth and division. Reduced RPS14 expression at 72 h in HCMV-infected emetine-treated cells was a result of its degradation, which was consequential to its interaction with MDM2. The reported interaction of RPS14 with MDM2 [21] directed us to investigate the role of RPS14 in the setting of MDM2-p53 interaction in HCMV replication (model, Fig 11). Emetine treatment improved the interaction between RPS14 and MDM2 in infected high-density cells. In non-infected cells (S4A Fig) the interaction of RPS14-MDM2 was not modified by emetine, likely because RPS14 did not translocate into the nucleus (S4B Fig). Therefore, infection facilitates nuclear translocation of RPS14 by emetine.

In infected cells emetine induced RPS14 translocation into the nucleus (Fig 6A), where it could interact with MDM2 and compete with viral proteins, such as IE2 (S5 Fig) and cellular proteins such as p53 on the acidic domain of MDM2 (Fig 9C and 9D). Although the binding site of MDM2-IE2 has not been characterized, it is possible that RPS14 bound MDM2 is incapable of binding to IE2. Later during infection, RPS14 relocalized to the cytoplasm, was ubiquitinated and degraded (Figs 6B, 8A & 8B). RPS14 interacted with MDM2 at 24 hpi (Fig 5C), but at this time point its expression level was similar in both low and high cell density (Fig 5A), suggesting that a balance between virus-induced RPS14 and its emetine-triggered degradation was still maintained at this time point. However, at 72 hpi, when virus replication was sufficiently inhibited, a significant reduction in the expression of RPS14 was observed in emetine-treated condition, indicating emetine-mediated RPS14 degradation (Fig 5A).

The dependence of emetine activity against HCMV on cell density (Fig 4) suggested that interaction of RPS14 with cell cycle regulators MDM2 and p53 may contribute to its activities. Reduced MDM2 expression during HCMV infection has been reported [26], and also shown here at 72 hpi (Fig 9A). Although infection at MOI 2–5 was reported to induce p53 expression, albeit reduction in p53 activity [32–35], at MOI 1 we did not observe changes in p53 expression. Emetine treatment resulted in increased p53 activity in infected cells, evidenced by p21 mRNA expression (Fig 9B). Levels of MDM2 and p53 were stabilized with emetine (Fig 9A and 9C), suggesting their interaction might be modified by the drug. Our data reveal that while the MDM2-p53 interaction is induced by HCMV, emetine disrupts it by 24 h, resulting in stabilization of each interacting partner (Fig 9D). In infected low-density cells in which HCMV escaped from emetine suppression (Fig 4), MDM2-p53 interaction was not disrupted by the drug (Fig 9D and 9E). Notably, in non-infected low-density cells the interaction between MDM2 and p53 was more significant than in high-density cells, suggesting that emetine fails to disrupt pre-existing interaction between MDM2-p53 in the low density cells (Fig 9D and 9E) [36,37].

The p53 protein binds to MDM2 at the acidic domain (amino acid residues 235–300) [23]. RPS14 also binds to the same acidic domain of MDM2 at an overlapping region with the p53 binding site (residues 215–300) [21]. Therefore, RPS14 may be competing with p53 for the same binding domain on MDM2. The reported mutations in the C-terminus domain of RPS14 that lead to emetine resistance; Arg-149-Cys and Arg-150-His [38], may imply altered binding to MDM2. Alternatively, haploinsufficiency of RPS14 may affect the interaction of MDM2-p53 [39]. Similarly, our RPS14 knockdown data indicate its necessity for the anti-viral activity of emetine, and that this activity affects the interaction of MDM2-p53 (Fig 10).

In HCMV-infected cells, binding of RPS14 to MDM2 was required for emetine activities. In low-density cells, MDM2 and p53 already interacted, suggesting the acidic domain of MDM2 was already occupied by p53. Therefore, RPS14 could not bind to MDM2, resulting in loss of emetine activity against HCMV. In contrast, in high-density cells, emetine induced RPS14 binding to the free MDM2-acidic domain and prevented virus-mediated interaction between MDM2-p53, resulting in stabilization of p53 and MDM2. These findings were also supported by the early activity of emetine (Fig 2B). If added after 12 hpi, emetine failed to inhibit HCMV, since by that time HCMV may engage p53 with MDM2, resulting in blocking of the RPS14 binding site on MDM2. Although in non-infected cells, emetine stabilized MDM2 and p53 in both cell densities (Fig 9C), it could not induce the nuclear translocation of RPS14 or its binding with MDM2 (S4 Fig). Future studies will address the consequences of disruption of MDM2-p53 binding and stabilization of p53 and MDM2 as a cellular strategy for HCMV inhibition.

Our results show efficient inhibition of HCMV replication *in vitro* and MCMV replication *in vivo*, suggesting that repurposing of emetine at a much lower dose may provide therapeutic/prophylactic strategy for HCMV through a host-directed antiviral mechanism. Although the route of administration and potential cumulative toxicities must be tested, the doses required for virus inhibition are substantially lower than the traditional emetine doses that have been used in the past. For amebiasis, emetine has been administered daily at 1 mg/kg for up to 10 days (maximal dose of 600 mg). Severe side effects occurred rarely and were only observed at high doses. Emetine was well-tolerated when delivered intravenously at 1.5 mg/kg doses twice a week in clinical trials as an anti-tumor agent [40]. Patients treated with 1 mg/kg emetine daily for 10 days did not experience any toxicity [41]. We show that MCMV replication was inhibited using an oral dose of 0.1 mg/kg. Using allometric scaling this translates to a human dose of 0.008 mg/kg [42]. Based on our PK data, emetine can be administered every 3 days. For a 60 kg individual 0.5 mg/dose would be considered for HCMV therapy. In one month, 10 doses will result in a cumulative dose of 5 mg. Therefore, to reach the 600 mg amebiasis dose 120 months of HCMV therapy would need to be provided. The expected cumulative dose with regimens used in general for HCMV therapy would be substantially lower than the doses that have been used in the past. The therapeutic plasma concentration of emetine is 0.005–0.075 μg/mL [43]. Our *in vitro* data suggest that at therapeutic plasma concentration HCMV replication can be fully inhibited. In addition, our PK studies support wide and prolonged tissue distribution, which may be an important factor for HCMV inhibition. Although emetine displays activities against other intracellular pathogens [44–49], its mode of action has been largely unknown. The killing of *Entamoeba histolytica* was attributed to inhibition of protein synthesis, an activity that is distinct from HCMV inhibition. Our study provides evidence for use of an old agent with distinct cellular activities resulting in HCMV inhibition.

## Materials and Methods

### Ethics statement

Animal work was carried out in strict accordance with the recommendations in the Guide for the Care and Use of Laboratory Animals of the National Institutes of Health. The animal protocol (protocol number MO13M296) was approved by the Institutional Animal Care and Use Committee (IACUC) of Johns Hopkins University.

### Compounds

Ganciclovir (GCV), MG132, CPG 2006, cycloheximide and emetine dihydrochloride hydrate were purchased from Sigma-Aldrich (St. Louis, MO).

### Viruses

The pp28-luciferase HCMV Towne and a GCV-resistant pp28-luciferase were used [50,51]. The recombinant viruses express luciferase under the control of the late CMV gene promoter, pp28. Luciferase expression is activated 48–72 hours post infection (hpi). The recombinant viruses provide a highly-sensitive reporter system which correlates with plaque reduction assay [50]. The Towne HCMV strain (ATCC VR-977) was used for plaque reduction, quantitative reverse transcriptase PCR (qRT-PCR), and immunoprecipitation assays. Human herpes virus strains were: luciferase HSV1- KOS/Dlux/oriS [52] and clinical isolates of HSV2. The clinical isolates were provided by the clinical microbiology laboratory with no identifiers that can link to a specific subject. Murid Herpesvirus (MCMV: ATCC VR-1399) was used for infection of mouse embryonic fibroblasts (MEFs) and mice.

### Cell culture, virus infection and anti-viral assays

Human Foreskin Fibroblasts (HFFs) passage 12–16 (ATCC, CRL-2088) and mouse embryonic fibroblasts (MEFs) passage 9–14 (ATCC, CRL-1658) were grown in Dulbecco's Modified Eagle Medium (DMEM) containing 10% fetal bovine serum (FBS) (Gibco, Carlsbad, CA) in a 5% CO_2_ incubator at 37°C. Infection with HCMV or HSV was performed in HFFs, and infection with mouse CMV was performed in MEFs. For HCMV, cells were seeded in either a 96-well plate or 100 mm culture plates (Corning Costar, Sigma Aldrich) at 0.5 or 2 million cells /plate. Following 90 minute adsorption, media was removed and cells were washed with PBS. Media containing 4% FBS and compounds were added to each well. Infected treated HFFs were collected at 72 hpi and lysates were assayed for luciferase activity using a luciferase assay kit (Promega, Madison, WI) on GloMax-Multi+ Detection System (Promega). In second cycle assays, supernatants were collected from all conditions of the first cycle at 96 hpi and used for infection of fresh HFFs in 96-well plates. Luciferase activity was measured 72 h following second cycle infection. For HSV1-KOS/Dlux/oriS, a luciferase assay was performed at 24 hpi. Plaque assays were performed with clinical isolates of HSV2 and HCMV Towne (ATCC VR-977). HFFs were seeded at 3 million cells/plate in 12-well plates and infected 24 h later with HSV2 at 200 PFU/well. For HCMV or MCMV, HFFs or MEFs were seeded at 0.5 or 2 million cells /plate in a 12-well plate and infected 24 hours later at 100 PFU/well. Following virus adsorption (60 min and 90 min for HSV and HCMV, respectively), virus was aspirated, and DMEM containing 4% fetal bovine serum (FBS) with (for HSV) or without (for HCMV/MCMV) 0.5% carboxymethyl-cellulose, were added with the compounds at indicated concentrations into triplicate wells. After incubation at 37°C for 10 days (for HCMV), 3 days for MCMV or 2 days (for HSV), the overlay was removed and plaques were counted after crystal violet staining.

### Generation of drug resistant virus

Screening of drug resistant virus was performed as reported [53]. Two million cells were plated on a 6-well plate and infected with HCMV (MOI 0.05). After 90 min cells were washed with PBS and 10 nM of emetine or 0.5 μM of GCV were added. The cells were maintained in DMEM with 4% FBS until plaques were observed. The supernatants from these plates were used to infect fresh HFFs in a 6-well plate and each time drug concentration was increased by two fold. The cells were passaged 5 times until a final drug concentration of emetine (2.4 μM) and GCV (10.5 μM). DNA extracted from supernatants collected at the last stage was used for UL97 sequencing.

### Cell viability

Cells were seeded in 96-well plates, treated with various concentrations of emetine and incubated at 37°C for 3 days. Cell viability was determined by an MTT-based colorimetric assay (Sigma-Aldrich), and performed at the same time points as the antiviral assay.

### Drug combination

The combined inhibitory effect of compounds on HCMV replication was determined in infected HFFs as previously reported [54]. The Bliss model, in which drug combination represents the product of two probabilistically independent events, was used for analysis [55,56].

### Translation inhibition assay for emetine

One million HFFs were seeded in a 96-well black, clear bottom tissue culture plate. After 24 h, cells were infected with HCMV Towne and treated with 50, 100, 1000, 2000 and 5000 nM emetine for 24 h or 100 mM cycloheximide for 30 minutes. A protein synthesis assay kit (Cayman Chemical, Ann Arbor, Michigan) was used according to manufacturer's protocol to quantify emetine-mediated translation inhibition using a fluorescence based assay [57].

### Add-on and removal studies

HFFs were infected with HCMV Towne, and at 0, 6, 12, 24, 36 and 48 hpi, emetine or GCV were added. For time-of-removal studies, medium containing the compounds was removed at 0, 6, 12, 24, 36 and 48 hpi, cells were washed three times with PBS, and drug-free medium was added. Culture supernatants were collected at 72 hpi and titration of infectious virus was performed after 14 days by plaque assay.

### Immunofluorescence

Emetine, GCV and a human-specific Toll-like receptor 9 (TLR9) ligand, CpG 2006 [58], were used to determine inhibition of virus entry. Compounds were diluted in serum-free medium and added to HFFs seeded on chamber slides 24 h prior to infection. After infection and treatment, cells were fixed, permeabilized, and air-dried. Cells were incubated with mouse monoclonal anti-pp65 antibody at 37°C in humidified chambers for 1 h, washed three times with 0.1% Tween 20 in PBS (PBST), incubated with rhodamine conjugated anti-mouse IgG (Sigma Chemical Co) at 37°C in humidified chambers for 1 h, and washed with PBST (0.1% Tween 20). A drop of mount oil containing DAPI (4,6-diamidino-2-phenylindole) (Santa Cruz) was added to the slides before visualization with a Nikon Eclipse E-800 fluorescence microscope.

### Pharmacokinetics (PK) and CMV inhibition in mice

Male BALB/c mice (n = 3 per time point) were treated with a single oral administration of 0.1 mg/kg emetine. The dosing solution was prepared in saline with a dosing volume of 10 mL/kg. Blood and tissue samples including liver, lung and spleen were collected at 0, 0.083, 0.25, 0.5, 0.75, 1, 2, 3, 4, 7, 24, 30, 48, 72 and 96 hr. Emetine concentration in plasma and tissue homogenates was determined by LC-MS/MS. Pharmacokinetic parameters (C_max_, T_max_, AUC and t_1/2_) were calculated with a non-compartmental approach using the Pharsight WinNonLin software (Ver. 6.4). The experimental procedures were approved by the Animal Care and Use Committee of Division of Veterinary Resources, NIH.

For infection experiments BALB/c mice, 4–6 weeks old, were purchased from Harlan Laboratories (Indianapolis, Indiana). The Animal Care and Use Committee of Johns Hopkins University approved the experimental procedures. After 2–3 days of adaptation to the housing environment, mice were randomly divided into seven groups as follows: control (5 mice), infected (12 mice), infected + emetine, 0.1 mg/kg treated at 72 hpi (10 mice), infected + emetine 1.0 mg/kg (10 mice) treated at 72 hpi, infected + emetine, 0.1 mg/kg treated at 24 hpi (8 mice), infected + emetine 1.0 mg/kg (8 mice) treated at 24 hpi and infected + GCV 10 mg/kg (10 mice). Mice were infected intraperitoneally with 10^6^ PFU/mice (0.1 mL in 0.8% saline). Control mice received 0.1 ml of saline intraperitoneally. Emetine was administered orally every three days. GCV was administered intraperitoneally twice daily. A total of three doses of emetine and ten doses of GCV was administered. Control and infected mice received equivalent volumes of saline. Mice were sacrificed at day 14 after infection. Blood samples were collected by cardiac puncture. Salivary glands and liver were harvested and stored at -80°C. Organs were homogenized in DMEM with 4% FBS at a final concentration of 100 mg/mL. Two million MEFs were seeded into 24-well plates. From each sample, 5% of the salivary gland homogenate or 10% of the liver homogenate was used for infection of MEFs in triplicates. Plaques were counted after three days. Whole blood viral load was measured at day 14 by real-time PCR of the glycoprotein B (gB) gene [59]. DNA was extracted using the DNA blood mini kit (Qiagen, Georgetown, MD).

### RNA isolation and real-time quantitative reverse transcriptase (qRT) PCR

Total RNA was isolated from cultured cells using RNeasy Mini kit (Qiagen). RevertAid first strand cDNA synthesis kit (Fermentas life sciences, Cromwell Park, MD) was used to synthesize first strand cDNA from total RNA using oligo-dT primers. Negative RT reactions were included to ensure the specificity of qRT-PCR reactions. Synthesis of first strand cDNA from mRNA template was carried out at 42°C for 1 h. Quantitative RT-PCR was performed in triplicates using specific primers for p21 (F: 5’ TGG AGA CTC TCA GGG TCG AAA 3’; R: 5’ CGG CGT TTG GAG TGG TAG AA 3’) and SYBR green (Fermentas life science) with two-step cycling protocol (95°C for 15 s, 60°C for 1 min). GAPDH (F: 5’ CGG AGT CAA CGG ATT TGG TCG TAT 3’; R: 5’ AGC CTT CTC CAT GGT GGT GAA GAC 3’) was used as the internal control.

### SDS-polyacrylamide gel electrophoresis and immunoblot analysis

Cell lysates were quantified for protein content using bicinchoninic acid (BCA) protein assay kit (Pierce Chemical, Rockford, IL). Equivalent amount of proteins were used for Western blot analysis as described previously (11). Protein bands were visualized by chemiluminescence using Western Blotting Luminol Reagent (Santa Cruz Biotechnology, Santa Cruz, CA). The following antibodies were used for detection of HCMV proteins: mouse anti-IE1 and IE2, (MAb810); mouse anti-UL44 (Santa Cruz biotechnology); mouse monoclonal anti-pp65 (Vector laboratories, Burlingame, CA); mouse monoclonal anti-β-actin antibody (Millipore, Billerica, MA); rabbit polyclonal anti-RPS14 and mouse monoclonal anti-MDM2 (AbCam, Cambridge, UK), mouse monoclonal anti-p53 (Santa Cruz); horseradish peroxidase (HRP)-conjugated goat anti-rabbit IgG (Cell Signaling); and HRP-conjugated sheep anti-mouse IgG, (GE Healthcare, Waukesha, WI). For detection of co-immunoprecipitated proteins, protein A-HRP conjugate (Cell Signaling Technologies, Beverly, MA) was used as secondary antibody to eliminate the interfering IgG bands [60].

### Transient transfections

HEK-293 cells seeded into 10 cm petri dishes were transfected with HCMV plasmid encoding IE1 and IE2 (pRL45) [61], using Lipofectamine 2000 in serum free medium. After 6 h, 10% FBS containing media was added along with 10 μM of MG132. Following overnight incubation emetine (75 nM) or GCV (5 μM) were added for 4 h. Cells were then harvested to prepare lysates for IP.

### Co-immunoprecipitation (co-IP) and immunoblotting

Non-infected, infected or emetine-treated HFFs were treated with MG132 (10 μM) for 12h. Cells were harvested after 24 hpi and lysed with IP buffer containing 150 mM NaCl, 50mM Tris pH 7.5, 2 mM EDTA, 0.5% TritonX-100 and 0.5% NP-40-40. 1 mg of lysate was precleared with bead slurry for 30 min. The precleared lysates were incubated with anti-MDM2 (2 μg) antibody overnight. The antibody complexes were isolated using protein A/G beads (Santa Cruz), washed three times with 50% IP buffer. The immunoprecipitate-protein A/G beads were boiled in SDS sample buffer, and the supernatant was analyzed on SDS-PAGE gels after immunoblotting as described previously. 1% of the cell lysate used for IP was loaded on gels as ‘Input’. Confirmatory reverse IPs were performed as described in each experiment. Isotype control antibodies included: rabbit IgG polyclonal, mouse IgG2a kappa and mouse IgG2b kappa monoclonal (Abcam). Mouse monoclonal RPS19 antibody (Abcam) served as an additional negative control for the IPs. Densitometry analysis was performed to determine relative ratio of immunoprecipitated protein to its input level.

### Confocal microscopy

Two million cells were plated on a chamber slide followed by infection with HCMV and treatment with emetine or GCV for 24 or 72 h. Cells were fixed with 3.7% paraformaldehyde for 20 min at room temperature, permeabilized with ice cold methanol for 10 min at -20°C and blocked with 5% bovine serum albumin in 0.5% Tween-20 for 20 min at room temperature. Cells were incubated with primary antibodies at 4°C overnight, washed and incubated with fluorescently-labeled secondary antibodies for 2 h at 37°C. Fluorescence microscopy was performed using a confocal laser scanning microscope (Nikon EZ C1). All images were captured at 60X magnification and processed under identical conditions with constant parameters (including scan speed and excitation and emission wavelengths) using Nikon EZ C1 software. Data analysis (percent nuclear localization) was performed by NIS-Elements software (Nikon) for a minimum of 40 cells in the high-density and 25 cells in the low-density samples from two fields per condition.

### RPS14 degradation

HFFs were plated at 0.5 or 2 million/plate in 6-well plates, Cells were infected and either untreated or treated with emetine (75 nM) or GCV (5 μM). At 24 hpi cells were treated with cycloheximide (100 μg/mL). Cell lysates were collected for RPS14 Western blot analysis at 0, 15 min, 1 h, 2 h, 4 h and 8 h post cycloheximide.

### Lentivirus-mediated knockdown (KD) of RPS14

Human TRC lentiviral shRNA constructs (Sigma-Aldrich) were used for RPS14 knockdown (KD) in HFFs. Multiple validated shRNAs (Clone ID: TRCN0000008641-4) targeting different regions of RPS14 mRNA were used to generate stable cell lines [62,63]. TRC non-targeting control plasmid was used to rule out non-specific effects of shRNA constructs. Individual shRNA constructs were packaged using lentivirus as described [64,65]. Lentivirus particles containing shRNA were transduced into HFFs. Puromycin (2 μg/ml) was added to select for stably transduced cells. Control HFFs and RPS14 KD HFFs were counted and equal number of cells was plated prior to infection or treatment.

### Statistical analysis

Statistical significance was assessed with GraphPad Prism 5.0 software. Data are presented as mean ± standard deviation, SD (n ≥ 3). Student’s t test was used to determine whether the mean of two groups are significantly different. In all analyses, two sided *p* values were used, and *p*<0.05 was considered statistically significant. For animal studies, one way Anova was used to determine significance. Densitometry analysis was performed with ImageJ.

## Supporting Information

S1 Table EC_50_ of emetine against different human herpesviruses and MCMV. (DOCX)

S2 Table PK data of emetine concentrations in the tested organs. (DOCX)

S1 Fig Low concentrations of Emetine do not inhibit protein synthesis in non-infected or HCMV-infected HFFs. One million cells were seeded in a 96 well black, clear bottom plate and mock or HCMV-infected (Towne) followed by treatment with the indicated doses of emetine for 24 h or CHX for 30 minutes. Puromycin analog O-Propargyl-puromycin (OPP) was added for termination of polypeptides. Protein synthesis was quantified as indicated in the materials and methods section. **A)** Cells were visualized using Nikon Eclipse E-800 fluorescence microscope. **B)** Fluorescence was measured using a filter to detect FITC (excitation/emission = 485/535 nm). Results are shown as the mean of the percent fluorescence intensities compared to non-emetine-treated control ± SD.
(TIF)

S2 Fig Specificity of protein interaction using anti-RPS19 and isotype control antibodies. HFFs were seeded at 2 million/plate in 100 mm dishes, infected with Towne followed by treatment with emetine (75 nM) or GCV (5μM) for 24h. MG132 (10 μM) was added after 12 h. At 24 hpi, lysates were collected and subjected to IP with **A)** rabbit IgG isotype control followed by immunoblotting with anti-RPS14. IP with anti-RPS14 antibody were used as a positive control. **B)** mouse IgG-2a isotype control followed by immunoblotting with anti-MDM2 or anti-p53 antibody. IP with anti-MDM2 or anti-p53 antibody were used as a positive control. **C)** anti-RPS19 antibody followed by immunoblotting with anti-MDM2. Mouse IgG-2b was used as an isotype control.
(TIF)

S3 Fig Emetine induces RPS14 and MDM2 interaction in MCMV-infected MEFs and disrupts the interaction between MDM2 and p53. Cells were seeded at 2 million/plate in 100 mm dishes, infected with MCMV followed by treatment with emetine (75 nM) or GCV (5μM) for 6h. MG132 (10 μM) was added after 2h. At 6 hpi, lysates were collected and subjected to IP with **A)** anti-MDM2 followed by immunoblotting with anti-RPS14 antibody (upper panel). In reverse reaction, IP was performed with anti-RPS14 followed by immunoblotting with anti-MDM2 antibody (lower panel). **B)** anti-MDM2 antibody followed by immunoblotting with anti-p53 antibody (upper panel) or IP with anti-p53 antibody followed by immunoblotting with anti-MDM2 antibody (lower panel). **C)** Inputs from each lysate were detected for MDM2, p53 and RPS14 content.
(TIF)

S4 Fig RPS14 does not interact with MDM2 in non-infected emetine treated cells and is not localized in the nuclear compartment. **A)** Cells were seeded at 2 or 1 million/plate in 100 mm dishes and treated emetine (75 nM) or GCV (5 μM) for 24 h. MG132 (10 μM) was added after 12 h. Lysates were collected at 24 h and IP was performed with anti-MDM2 antibody followed by immunoblotting with anti-RPS14 antibody. **B)** Cells were seeded at 2 million/plate in a 4-well chamber slide, and treated with emetine (75 nM) or GCV (5 μM) for 72 h. Cells were stained with IE1/2 (Alexa 555:Red) and RPS14 (FITC: Green) and nuclear DAPI. Stained slides were subjected to confocal microscopy and colocalization was quantified using NIS elements.
(TIF)

S5 Fig Emetine disrupts MDM2-IE2 interaction. **A**) HEK293 cells were seeded in 100 mm dishes and transfected with pRL45 plasmid, followed by treatment with MG132 (10 μM) for 12h. Emetine (75 nM) or GCV (5 μM) were then added for 4h. An IP was performed with anti- IE1/IE2 antibody followed by immunoblotting with anti-MDM2 antibody or **B**) Reverse IP was performed with anti-MDM2 antibody followed by immunoblotting with anti-IE1/IE2 antibody.
(TIF)

## References

[1] Griffiths PD, Clark DA, Emery VC (2000) Betaherpesviruses in transplant recipients. J Antimicrob Chemother 45 Suppl T3: 29–34. 10855769 10.1093/jac/45.suppl_4.29

[2] Kovacs A, Schluchter M, Easley K, Demmler G, Shearer W et al (1999) Cytomegalovirus infection and HIV-1 disease progression in infants born to HIV-1-infected women. Pediatric Pulmonary and Cardiovascular Complications of Vertically Transmitted HIV Infection Study Group. N Engl J Med 341: 77–84. 10395631 10.1056/NEJM199907083410203 PMC4280563

[3] Demmler GJ (1991) Infectious Diseases Society of America and Centers for Disease Control. Summary of a workshop on surveillance for congenital cytomegalovirus disease. Rev Infect Dis 13: 315–329. 1645882 10.1093/clinids/13.2.315

[4] Manicklal S, Emery VC, Lazzarotto T, Boppana SB, Gupta RK (2013) The "silent" global burden of congenital cytomegalovirus. Clin Microbiol Rev 26: 86–102. 26/1/86 [pii]; 10.1128/CMR.00062-12 23297260 PMC3553672

[5] Prichard MN, Kern ER (2011) The search for new therapies for human cytomegalovirus infections. Virus Res 157: 212–221. 10.1016/j.virusres.2010.11.004 21095209 PMC3068221

[6] Schreiber A, Harter G, Schubert A, Bunjes D, Mertens T et al (2009) Antiviral treatment of cytomegalovirus infection and resistant strains. Expert Opin Pharmacother 10: 191–209. 10.1517/14656560802678138 19236193

[7] Steininger C (2007) Novel therapies for cytomegalovirus disease. Recent Pat Antiinfect Drug Discov 2: 53–72. 18221163 10.2174/157489107779561634

[8] Kimberlin DW, Lin CY, Sanchez PJ, Demmler GJ, Dankner W et al (2003) Effect of ganciclovir therapy on hearing in symptomatic congenital cytomegalovirus disease involving the central nervous system: a randomized, controlled trial. J Pediatr 143: 16–25. 12915819 10.1016/s0022-3476(03)00192-6

[9] Kimberlin DW, Jester PM, Sanchez PJ, Ahmed A, Arav-Boger R et al (2015) Valganciclovir for symptomatic congenital cytomegalovirus disease. N Engl J Med 372: 933–943. 10.1056/NEJMoa1404599 25738669 PMC4401811

[10] Jabs DA, Martin BK, Forman MS (2010) Mortality associated with resistant cytomegalovirus among patients with cytomegalovirus retinitis and AIDS. Ophthalmology 117: 128–132. 10.1016/j.ophtha.2009.06.016 19818505 PMC2815221

[11] Chou SW (2001) Cytomegalovirus drug resistance and clinical implications. Transpl Infect Dis 3 Suppl 2: 20–24. 11926745 10.1034/j.1399-3062.2001.00004.x

[12] Krosky PM, Baek MC, Coen DM (2003) The human cytomegalovirus UL97 protein kinase, an antiviral drug target, is required at the stage of nuclear egress. J Virol 77: 905–914. 12502806 10.1128/JVI.77.2.905-914.2003 PMC140798

[13] Williams SL, Hartline CB, Kushner NL, Harden EA, Bidanset DJ et al (2003) In vitro activities of benzimidazole D- and L-ribonucleosides against herpesviruses. Antimicrob Agents Chemother 47: 2186–2192. 12821466 10.1128/AAC.47.7.2186-2192.2003 PMC161863

[14] Lischka P, Hewlett G, Wunberg T, Baumeister J, Paulsen D et al (2010) In vitro and in vivo activities of the novel anticytomegalovirus compound AIC246. Antimicrob Agents Chemother 54: 1290–1297. 10.1128/AAC.01596-09 20047911 PMC2826024

[15] Kaul DR, Stoelben S, Cober E, Ojo T, Sandusky E et al (2011) First report of successful treatment of multidrug-resistant cytomegalovirus disease with the novel anti-CMV compound AIC246. Am J Transplant 11: 1079–1084. 10.1111/j.1600-6143.2011.03530.x 21521474

[16] Winston DJ, Young JA, Pullarkat V, Papanicolaou GA, Vij R et al (2008) Maribavir prophylaxis for prevention of cytomegalovirus infection in allogeneic stem cell transplant recipients: a multicenter, randomized, double-blind, placebo-controlled, dose-ranging study. Blood 111: 5403–5410. 10.1182/blood-2007-11-121558 18285548 PMC5726327

[17] Shen L, Peterson S, Sedaghat AR, McMahon MA, Callender M et al (2008) Dose-response curve slope sets class-specific limits on inhibitory potential of anti-HIV drugs. Nat Med 14: 762–766. 10.1038/nm1777 18552857 PMC2743464

[18] Thoene JG, Lemons R, Boskovich S, Borysko K (1985) Inhibitors of protein synthesis also inhibit lysosomal proteolysis. Studies using cystinotic fibroblasts. J Clin Invest 75: 370–376. 3973010 10.1172/JCI111709 PMC423496

[19] Entner N, Grollman AP (1973) Inhibition of protein synthesis: a mechanism of amebicide action of emetine and other structurally related compounds. J Protozool 20: 160–163. 4347870 10.1111/j.1550-7408.1973.tb06025.x

[20] Madjar JJ, Nielsen-Smith K, Frahm M, Roufa DJ (1982) Emetine resistance in chinese hamster ovary cells is associated with an altered ribosomal protein S14 mRNA. Proc Natl Acad Sci U S A 79: 1003–1007. 6122207 10.1073/pnas.79.4.1003 PMC345887

[21] Zhou X, Hao Q, Liao J, Zhang Q, Lu H (2013) Ribosomal protein S14 unties the MDM2-p53 loop upon ribosomal stress. Oncogene 32: 388–396. 10.1038/onc.2012.63 22391559 PMC3736832

[22] Riley MF, Lozano G (2012) The Many Faces of MDM2 Binding Partners. Genes Cancer 3: 226–239. 10.1177/1947601912455322 23150756 PMC3494375

[23] Kulikov R, Winter M, Blattner C (2006) Binding of p53 to the central domain of Mdm2 is regulated by phosphorylation. J Biol Chem 281: 28575–28583. 16870621 10.1074/jbc.M513311200

[24] Moll UM, Petrenko O (2003) The MDM2-p53 interaction. Mol Cancer Res 1: 1001–1008. 14707283

[25] Worrall EG, Wawrzynow B, Worrall L, Walkinshaw M, Ball KL et al (2009) Regulation of the E3 ubiquitin ligase activity of MDM2 by an N-terminal pseudo-substrate motif. J Chem Biol 2: 113–129. 10.1007/s12154-009-0019-5 19568783 PMC2725272

[26] Zhang Z, Evers DL, McCarville JF, Dantonel JC, Huong SM et al (2006) Evidence that the human cytomegalovirus IE2-86 protein binds mdm2 and facilitates mdm2 degradation. J Virol 80: 3833–3843. 80/8/3833 [pii]; 10.1128/JVI.80.8.3833-3843.2006 16571800 PMC1440454

[27] Lurain NS, Chou S (2010) Antiviral drug resistance of human cytomegalovirus. Clin Microbiol Rev 23: 689–712. 10.1128/CMR.00009-10 20930070 PMC2952978

[28] Roy S, Arav-Boger R (2014) New cell-signaling pathways for controlling cytomegalovirus replication. Am J Transplant 14: 1249–1258. 10.1111/ajt.12725 24839861 PMC4280670

[29] Kern ER, Collins DJ, Wan WB, Beadle JR, Hostetler KY et al (2004) Oral treatment of murine cytomegalovirus infections with ether lipid esters of cidofovir. Antimicrob Agents Chemother 48: 3516–3522. 15328119 10.1128/AAC.48.9.3516-3522.2004 PMC514741

[30] Madjar JJ, Frahm M, McGill S, Roufa DJ (1983) Ribosomal protein S14 is altered by two-step emetine resistance mutations in Chinese hamster cells. Mol Cell Biol 3: 190–197. 6835209 10.1128/mcb.3.2.190-197.1983 PMC368521

[31] Browne EP, Wing B, Coleman D, Shenk T (2001) Altered cellular mRNA levels in human cytomegalovirus-infected fibroblasts: viral block to the accumulation of antiviral mRNAs. J Virol 75: 12319–12330. 11711622 10.1128/JVI.75.24.12319-12330.2001 PMC116128

[32] Hwang ES, Zhang Z, Cai H, Huang DY, Huong SM et al (2009) Human cytomegalovirus IE1-72 protein interacts with p53 and inhibits p53-dependent transactivation by a mechanism different from that of IE2-86 protein. J Virol 83: 12388–12398. JVI.00304-09 [pii]; 10.1128/JVI.00304-09 19776115 PMC2786713

[33] Casavant NC, Luo MH, Rosenke K, Winegardner T, Zurawska A et al (2006) Potential role for p53 in the permissive life cycle of human cytomegalovirus. J Virol 80: 8390–8401. 80/17/8390 [pii]; 10.1128/JVI.00505-06 16912290 PMC1563868

[34] Fortunato EA, Spector DH (1998) p53 and RPA are sequestered in viral replication centers in the nuclei of cells infected with human cytomegalovirus. J Virol 72: 2033–2039. 9499057 10.1128/jvi.72.3.2033-2039.1998 PMC109496

[35] Chen Z, Knutson E, Wang S, Martinez LA, Albrecht T (2007) Stabilization of p53 in human cytomegalovirus-initiated cells is associated with sequestration of HDM2 and decreased p53 ubiquitination. J Biol Chem 282: 29284–29295. M705349200 [pii]; 10.1074/jbc.M705349200 17698841

[36] Haupt Y, Maya R, Kazaz A, Oren M (1997) Mdm2 promotes the rapid degradation of p53. Nature 387: 296–299. 9153395 10.1038/387296a0

[37] Kubbutat MH, Jones SN, Vousden KH (1997) Regulation of p53 stability by Mdm2. Nature 387: 299–303. 9153396 10.1038/387299a0

[38] Diaz JJ, Roufa DJ (1992) Fine-structure map of the human ribosomal protein gene RPS14. Mol Cell Biol 12: 1680–1686. 1549121 10.1128/mcb.12.4.1680-1686.1992 PMC369611

[39] Boultwood J (2011) The role of haploinsufficiency of RPS14 and p53 activation in the molecular pathogenesis of the 5q- syndrome. Pediatr Rep 3 Suppl 2: e10 10.4081/pr.2011.s2.e10 pr.2011.s2.e10 [pii]. 22053272 PMC3206529

[40] Panettiere F, Coltman CA Jr. (1971) Experience with emetine hydrochloride (NSC 33669) as an antitumor agent. Cancer 27: 835–841. 4929936 10.1002/1097-0142(197104)27:4<835::aid-cncr2820270413>3.0.co;2-k

[41] Mastrangelo MJ, Grage TB, Bellet RE, Weiss AJ (1973) A phase I study of emetine hydrochloride (NSC 33669) in solid tumors. Cancer 31: 1170–1175. 4705155 10.1002/1097-0142(197305)31:5<1170::aid-cncr2820310520>3.0.co;2-4

[42] Reagan-Shaw S, Nihal M, Ahmad N (2008) Dose translation from animal to human studies revisited. FASEB J 22: 659–661. fj.07-9574LSF [pii]; 10.1096/fj.07-9574LSF 17942826

[43] Regenthal R, Krueger M, Koeppel C, Preiss R (1999) Drug levels: therapeutic and toxic serum/plasma concentrations of common drugs. J Clin Monit Comput 15: 529–544. 12578052 10.1023/a:1009935116877

[44] Knight R (1980) The chemotherapy of amoebiasis. J Antimicrob Chemother 6: 577–593. 6106010 10.1093/jac/6.5.577

[45] Muhammad I, Dunbar DC, Khan SI, Tekwani BL, Bedir E et al (2003) Antiparasitic alkaloids from Psychotria klugii. J Nat Prod 66: 962–967. 12880315 10.1021/np030086k

[46] Carney RW, Wojtkunski J, Konopka EA, deStevens G (1966) The chemical, spectral, and biological properties of monomethine cyanine dyes containing 1,3-benzoxazine and quinazoline nuclei. J Med Chem 9: 758–762. 4381839 10.1021/jm00323a027

[47] Rosenkranz V, Wink M (2008) Alkaloids induce programmed cell death in bloodstream forms of trypanosomes (Trypanosoma b. brucei). Molecules 13: 2462–2473. 10.3390/molecules13102462 18833031 PMC6244846

[48] Deng L, Dai P, Ciro A, Smee DF, Djaballah H et al (2007) Identification of novel antipoxviral agents: mitoxantrone inhibits vaccinia virus replication by blocking virion assembly. J Virol 81: 13392–13402. 17928345 10.1128/JVI.00770-07 PMC2168821

[49] Chaves Valadao AL, Abreu CM, Dias JZ, Arantes P, Verli H et al (2015) Natural Plant Alkaloid (Emetine) Inhibits HIV-1 Replication by Interfering with Reverse Transcriptase Activity. Molecules 20: 11474–11489. molecules200611474 [pii]; 10.3390/molecules200611474 26111177 PMC6272240

[50] He R, Sandford G, Hayward GS, Burns WH, Posner GH et al (2011) Recombinant Luciferase-Expressing Human Cytomegalovirus (CMV) for evaluation of CMV inhibitors. Virol J 8: 40 10.1186/1743-422X-8-40 21269468 PMC3041771

[51] Lurain NS, Chou S (2010) Antiviral drug resistance of human cytomegalovirus. Clin Microbiol Rev 23: 689–712. 10.1128/CMR.00009-10 20930070 PMC2952978

[52] Summers BC, Margolis TP, Leib DA (2001) Herpes simplex virus type 1 corneal infection results in periocular disease by zosteriform spread. J Virol 75: 5069–5075. 11333887 10.1128/JVI.75.11.5069-5075.2001 PMC114911

[53] Fischer L, Laib SK, Jahn G, Hamprecht K, Gohring K (2013) Generation and characterization of a GCV resistant HCMV UL97-mutation and a drug sensitive UL54-mutation. Antiviral Res 100: 575–577. S0166-3542(13)00286-6 [pii]; 10.1016/j.antiviral.2013.09.026 24120366

[54] Cai H, Kapoor A, He R, Venkatadri R, Forman M et al (2014) In vitro combination of anti-cytomegalovirus compounds acting through different targets: role of the slope parameter and insights into mechanisms of Action. Antimicrob Agents Chemother 58: 986–994. AAC.01972-13 [pii]; 10.1128/AAC.01972-13 24277030 PMC3910867

[55] Bliss C.I. (1939) The toxicity of poisons applied jointly. Ann Appl Biol 26: 585–615.

[56] Jilek BL, Zarr M, Sampah ME, Rabi SA, Bullen CK et al (2012) A quantitative basis for antiretroviral therapy for HIV-1 infection. Nat Med 18: 446–451. 10.1038/nm.2649 22344296 PMC3296892

[57] Liu J, Xu Y, Stoleru D, Salic A (2012) Imaging protein synthesis in cells and tissues with an alkyne analog of puromycin. Proc Natl Acad Sci U S A 109: 413–418. 1111561108 [pii]; 10.1073/pnas.1111561108 22160674 PMC3258597

[58] Iversen AC, Steinkjer B, Nilsen N, Bohnhorst J, Moen SH et al (2009) A proviral role for CpG in cytomegalovirus infection. J Immunol 182: 5672–5681. 10.4049/jimmunol.0801268 19380814

[59] Vliegen I, Herngreen S, Grauls G, Bruggeman C, Stassen F (2003) Improved detection and quantification of mouse cytomegalovirus by real-time PCR. Virus Res 98: 17–25. 14609626 10.1016/j.virusres.2003.08.009

[60] Lal A, Haynes SR, Gorospe M (2005) Clean Western blot signals from immunoprecipitated samples. Mol Cell Probes 19: 385–388. S0890-8508(05)00044-7 [pii]; 10.1016/j.mcp.2005.06.007 16146684 PMC1350844

[61] Pizzorno MC, O'Hare P, Sha L, LaFemina RL, Hayward GS (1988) trans-activation and autoregulation of gene expression by the immediate-early region 2 gene products of human cytomegalovirus. J Virol 62: 1167–1179. 2831379 10.1128/jvi.62.4.1167-1179.1988 PMC253124

[62] Ebert BL, Pretz J, Bosco J, Chang CY, Tamayo P et al (2008) Identification of RPS14 as a 5q- syndrome gene by RNA interference screen. Nature 451: 335–339. nature06494 [pii]; 10.1038/nature06494 18202658 PMC3771855

[63] Caceres G, McGraw K, Yip BH, Pellagatti A, Johnson J et al (2013) TP53 suppression promotes erythropoiesis in del(5q) MDS, suggesting a targeted therapeutic strategy in lenalidomide-resistant patients. Proc Natl Acad Sci U S A 110: 16127–16132. 1311055110 [pii]; 10.1073/pnas.1311055110 24043769 PMC3791697

[64] Tiscornia G, Singer O, Ikawa M, Verma IM (2003) A general method for gene knockdown in mice by using lentiviral vectors expressing small interfering RNA. Proc Natl Acad Sci U S A 100: 1844–1848. 12552109 10.1073/pnas.0437912100 PMC149921

[65] Kapoor A, Forman M, Arav-Boger R (2014) Activation of nucleotide oligomerization domain 2 (NOD2) by human cytomegalovirus initiates innate immune responses and restricts virus replication. PLoS One 9: e92704 10.1371/journal.pone.0092704 PONE-D-13-49464 [pii]. 24671169 PMC3966837

