# Supplementary material for: Efficacy and Mechanism of Action of Low Dose Emetine against Human Cytomegalovirus
Source: PLoS Pathog. 2016 Jun 23;12(6):e1005717. doi: 10.1371/journal.ppat.1005717 (PMC4919066; doi:10.1371/journal.ppat.1005717)
Supplement: S1 Table — (DOCX) [file ppat.1005717.s001.docx]

| Virus | Mean EC_50_ (μM) ± SD | Assay |
| --- | --- | --- |
| HCMV | 0.068 ± 0.00 | Plaque |
| MCMV | 0.036 ± 0.00 | Plaque |
| GCV-R | 0.038 ± 0.00 | Luciferase |
| HSV-1 | 0.056 ± 0.00 | Luciferase |
| HSV-2 | 0.033 ± 0.00 | Plaque |
