# Supplementary material for: Efficacy and Mechanism of Action of Low Dose Emetine against Human Cytomegalovirus
Source: PLoS Pathog. 2016 Jun 23;12(6):e1005717. doi: 10.1371/journal.ppat.1005717 (PMC4919066; doi:10.1371/journal.ppat.1005717)
Supplement: S2 Table — (DOCX) [file ppat.1005717.s002.docx]

|  | **LUNG** | | **LIVER** | | **SPLEEN** | |
| --- | --- | --- | --- | --- | --- | --- |
| **Time (h)** | Mean Conc. ng/g | Conc. μMol/kg | Mean Conc. ng/g | Conc. μMol/kg | Mean Conc. ng/g | Conc. μMol/kg |
|  |  |  |  |  |  |  |
| 0.083 | 1.07 | 0.002 | 3.59 | 0.007 |  |  |
| 0.25 | 1.49 | 0.003 | 6.18 | 0.013 |  |  |
| 0.5 | 2.90 | 0.006 | 17.7 | 0.037 | 1.12 | 0.002 |
| 0.75 | 4.89 | 0.010 | 37.5 | 0.078 | 3.12 | 0.006 |
| 1 | 8.11 | 0.017 | 56.5 | 0.117 | 5.60 | 0.012 |
| 2 | 9.70 | 0.020 | 92.2 | 0.192 | 8.81 | 0.018 |
| 3 | 17.9 | 0.037 | 152 | 0.316 | 15.3 | 0.032 |
| 4 | 34.2 | 0.071 | 210 | 0.437 | 30.6 | 0.064 |
| 7 | 46.6 | 0.097 | 253 | 0.527 | 44.9 | 0.093 |
| 24 | 66.1 | 0.138 | 93.0 | 0.193 | 84.3 | 0.175 |
| 30 | 64.1 | 0.133 | 83.6 | 0.174 | 92.5 | 0.193 |
| 48 | 37.4 | 0.078 | 34.9 | 0.073 | 66.6 | 0.139 |
| 72 | 23.7 | 0.049 | 21.2 | 0.044 | 45.2 | 0.094 |
| 96 | 14.3 | 0.030 | 13.5 | 0.028 | 28.1 | 0.058 |
