# Supplementary figures and images for: Efficacy and Mechanism of Action of Low Dose Emetine against Human Cytomegalovirus
Source: PLoS Pathog. 2016 Jun 23;12(6):e1005717. doi: 10.1371/journal.ppat.1005717 (PMC4919066; doi:10.1371/journal.ppat.1005717)

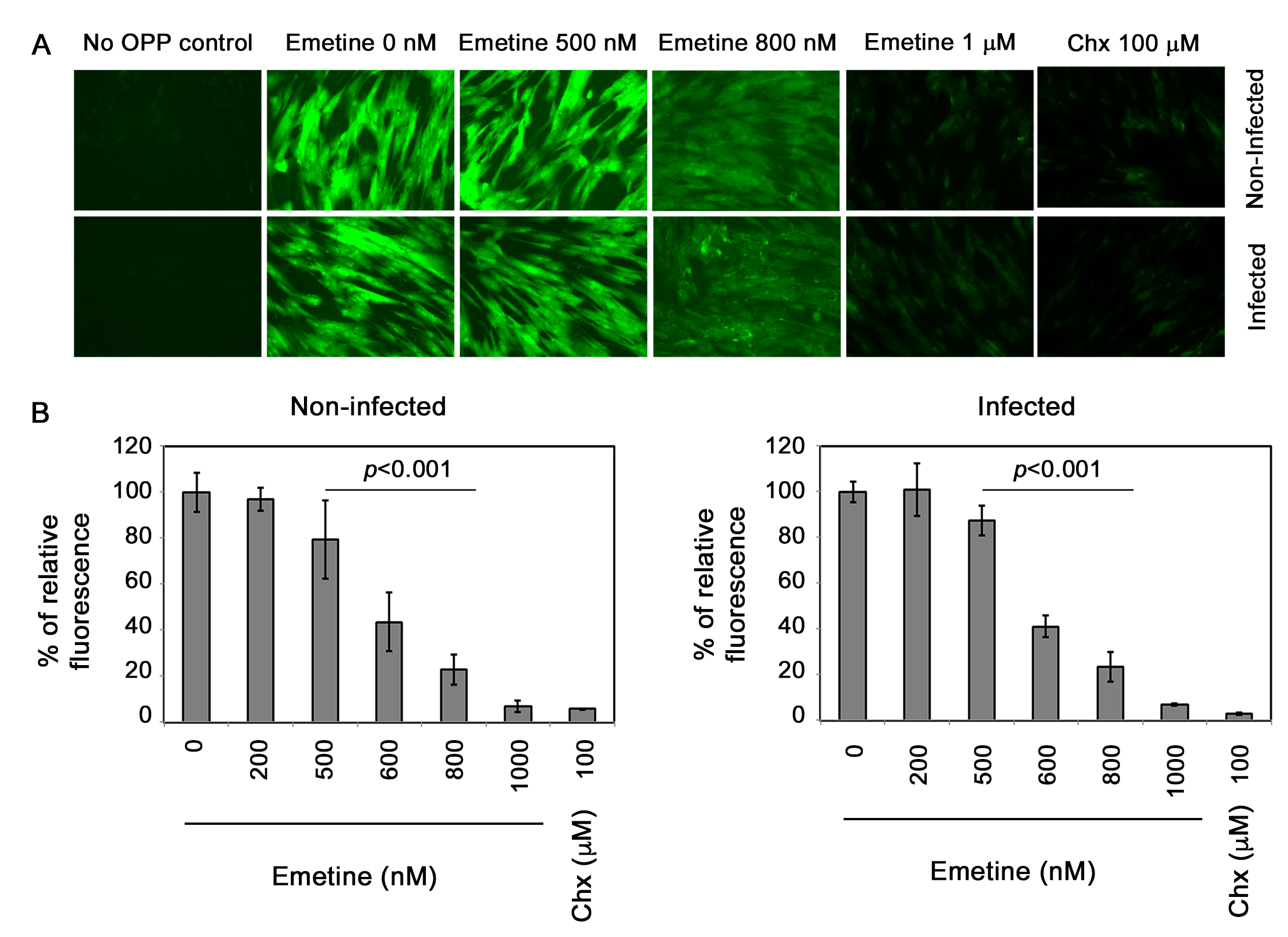

Supplement: S1 Fig — One million cells were seeded in a 96 well black, clear bottom plate and mock or HCMV-infected (Towne) followed by treatment with the indicated doses of emetine for 24 h or CHX for 30 minutes. Puromycin analog O-Propargyl-puromycin (OPP) was added for termination of polypeptides. Protein synthesis was quantified as indicated in the materials and methods section. A) Cells were visualized using Nikon Eclipse E-800 fluorescence microscope. B) Fluorescence was measured using a filter to detect FITC (excitation/emission = 485/535 nm). Results are shown as the mean of the percent fluorescence intensities compared to non-emetine-treated control ± SD. (TIF) [file ppat.1005717.s003.tif]

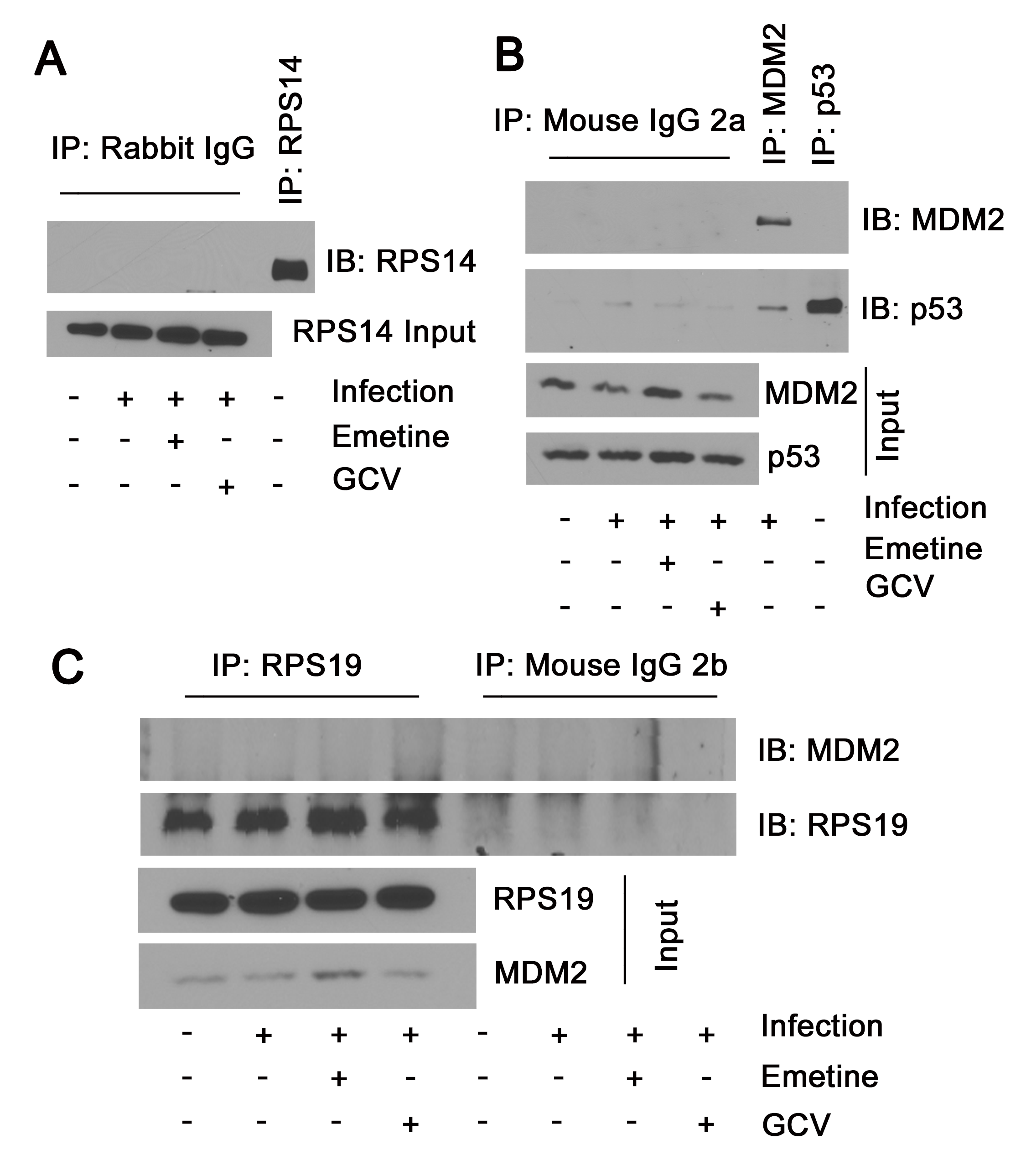

Supplement: S2 Fig — HFFs were seeded at 2 million/plate in 100 mm dishes, infected with Towne followed by treatment with emetine (75 nM) or GCV (5μM) for 24h. MG132 (10 μM) was added after 12 h. At 24 hpi, lysates were collected and subjected to IP with A) rabbit IgG isotype control followed by immunoblotting with anti-RPS14. IP with anti-RPS14 antibody were used as a positive control. B) mouse IgG-2a isotype control followed by immunoblotting with anti-MDM2 or anti-p53 antibody. IP with anti-MDM2 or anti-p53 antibody were used as a positive control. C) anti-RPS19 antibody followed by immunoblotting with anti-MDM2. Mouse IgG-2b was used as an isotype control. (TIF) [file ppat.1005717.s004.tif]

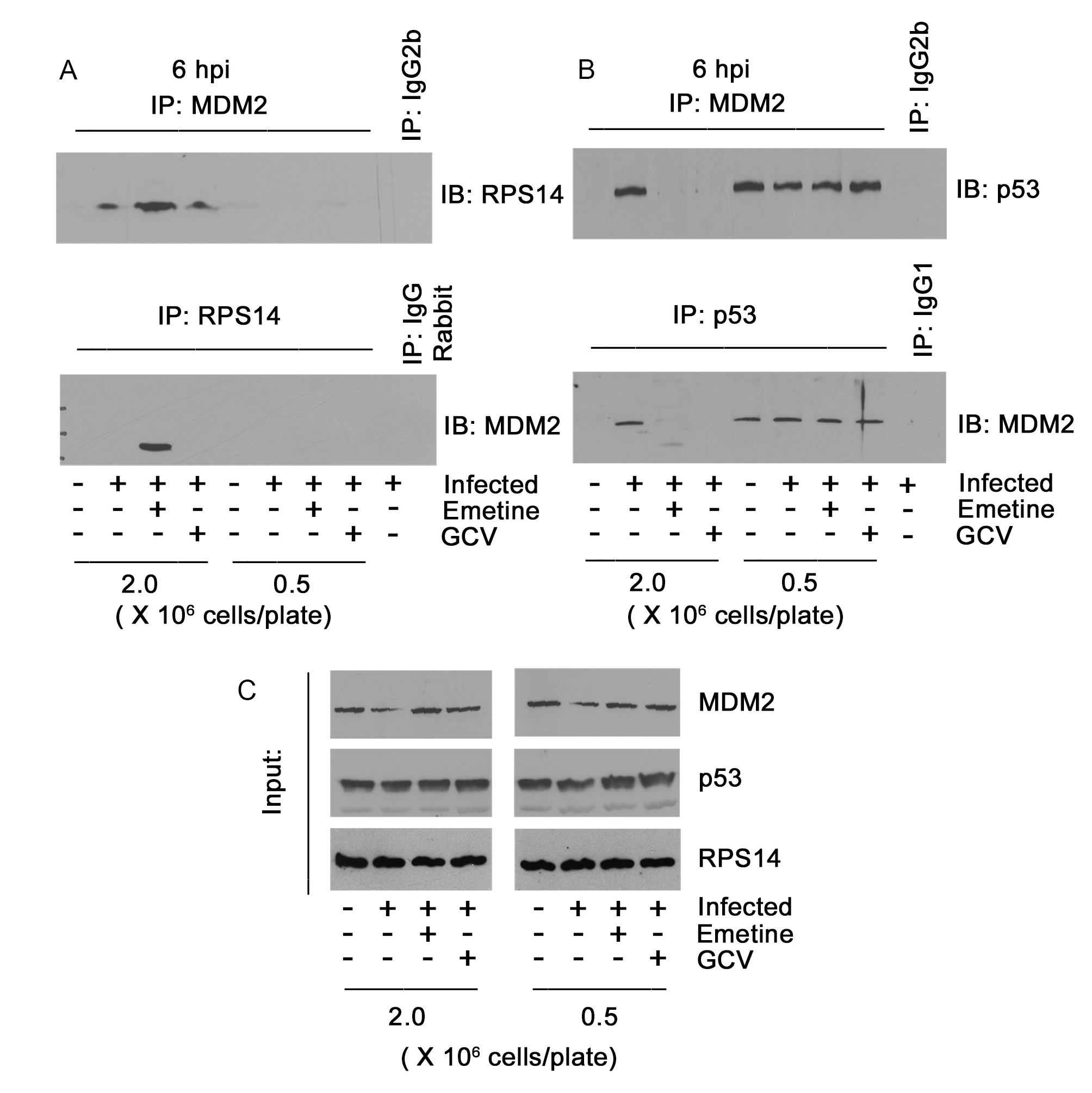

Supplement: S3 Fig — Cells were seeded at 2 million/plate in 100 mm dishes, infected with MCMV followed by treatment with emetine (75 nM) or GCV (5μM) for 6h. MG132 (10 μM) was added after 2h. At 6 hpi, lysates were collected and subjected to IP with A) anti-MDM2 followed by immunoblotting with anti-RPS14 antibody (upper panel). In reverse reaction, IP was performed with anti-RPS14 followed by immunoblotting with anti-MDM2 antibody (lower panel). B) anti-MDM2 antibody followed by immunoblotting with anti-p53 antibody (upper panel) or IP with anti-p53 antibody followed by immunoblotting with anti-MDM2 antibody (lower panel). C) Inputs from each lysate were detected for MDM2, p53 and RPS14 content. (TIF) [file ppat.1005717.s005.tif]

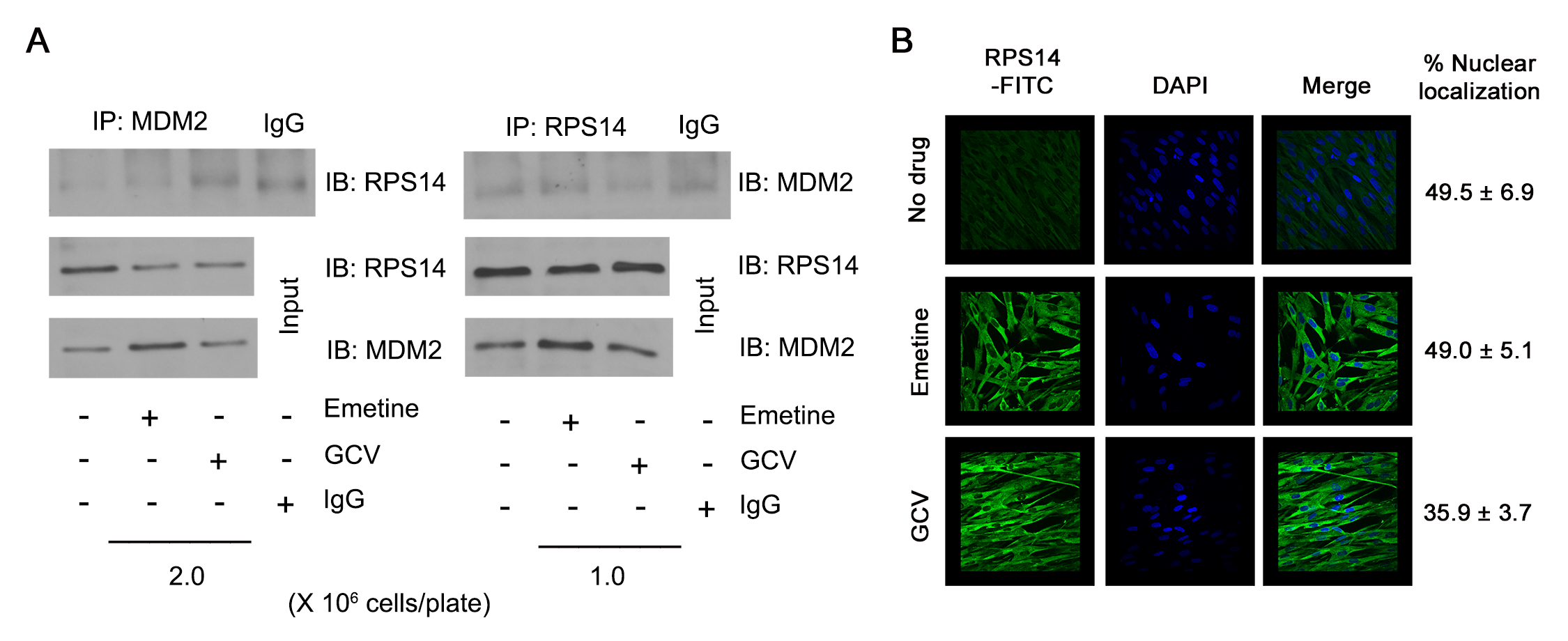

Supplement: S4 Fig — A) Cells were seeded at 2 or 1 million/plate in 100 mm dishes and treated emetine (75 nM) or GCV (5 μM) for 24 h. MG132 (10 μM) was added after 12 h. Lysates were collected at 24 h and IP was performed with anti-MDM2 antibody followed by immunoblotting with anti-RPS14 antibody. B) Cells were seeded at 2 million/plate in a 4-well chamber slide, and treated with emetine (75 nM) or GCV (5 μM) for 72 h. Cells were stained with IE1/2 (Alexa 555:Red) and RPS14 (FITC: Green) and nuclear DAPI. Stained slides were subjected to confocal microscopy and colocalization was quantified using NIS elements. (TIF) [file ppat.1005717.s006.tif]

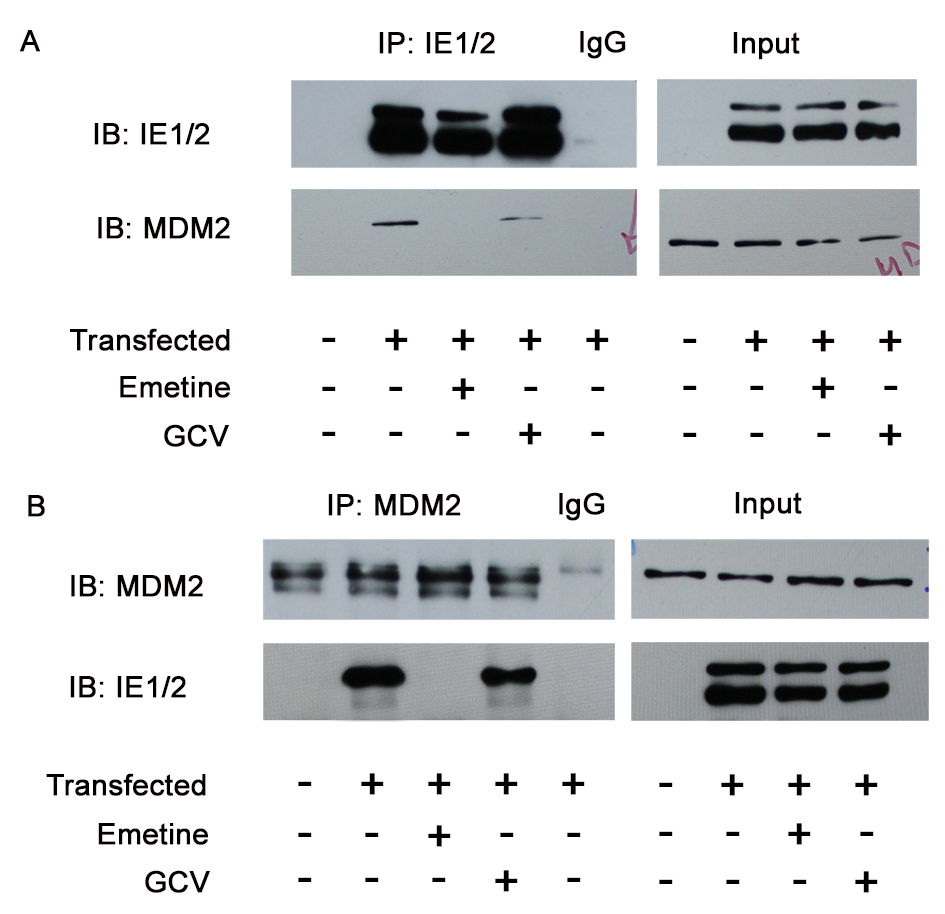

Supplement: S5 Fig — A) HEK293 cells were seeded in 100 mm dishes and transfected with pRL45 plasmid, followed by treatment with MG132 (10 μM) for 12h. Emetine (75 nM) or GCV (5 μM) were then added for 4h. An IP was performed with anti- IE1/IE2 antibody followed by immunoblotting with anti-MDM2 antibody or B) Reverse IP was performed with anti-MDM2 antibody followed by immunoblotting with anti-IE1/IE2 antibody. (TIF) [file ppat.1005717.s007.tif]
